# Detecting and Removing Inconsistencies between Experimental Data and Signaling Network Topologies Using Integer Linear Programming on Interaction Graphs

**DOI:** 10.1371/journal.pcbi.1003204

**Published:** 2013-09-05

**Authors:** Ioannis N. Melas, Regina Samaga, Leonidas G. Alexopoulos, Steffen Klamt

**Affiliations:** 1 National Technical University of Athens, Athens, Greece; 2 Max Planck Institute for Dynamics of Complex Technical Systems, Magdeburg, Germany; New York University, United States of America

## Abstract

Cross-referencing experimental data with our current knowledge of signaling network topologies is one central goal of mathematical modeling of cellular signal transduction networks. We present a new methodology for data-driven interrogation and training of signaling networks. While most published methods for signaling network inference operate on Bayesian, Boolean, or ODE models, our approach uses integer linear programming (ILP) on interaction graphs to encode constraints on the qualitative behavior of the nodes. These constraints are posed by the network topology and their formulation as ILP allows us to predict the possible qualitative changes (up, down, no effect) of the activation levels of the nodes for a given stimulus. We provide four basic operations to detect and remove inconsistencies between measurements and predicted behavior: (i) find a topology-consistent explanation for responses of signaling nodes measured in a stimulus-response experiment (if none exists, find the closest explanation); (ii) determine a minimal set of nodes that need to be corrected to make an inconsistent scenario consistent; (iii) determine the optimal subgraph of the given network topology which can best reflect measurements from a set of experimental scenarios; (iv) find possibly missing edges that would improve the consistency of the graph with respect to a set of experimental scenarios the most. We demonstrate the applicability of the proposed approach by interrogating a manually curated interaction graph model of EGFR/ErbB signaling against a library of high-throughput phosphoproteomic data measured in primary hepatocytes. Our methods detect interactions that are likely to be inactive in hepatocytes and provide suggestions for new interactions that, if included, would significantly improve the goodness of fit. Our framework is highly flexible and the underlying model requires only easily accessible biological knowledge. All related algorithms were implemented in a freely available toolbox *SigNetTrainer* making it an appealing approach for various applications.

## Author Summary

Cellular signal transduction is orchestrated by communication networks of signaling proteins commonly depicted on signaling pathway maps. However, each cell type may have distinct variants of signaling pathways, and wiring diagrams are often altered in disease states. The identification of truly active signaling topologies based on experimental data is therefore one key challenge in systems biology of cellular signaling. We present a new framework for training signaling networks based on interaction graphs (IG). In contrast to complex modeling formalisms, IG capture merely the known positive and negative edges between the components. This basic information, however, already sets hard constraints on the possible qualitative behaviors of the nodes when perturbing the network. Our approach uses Integer Linear Programming to encode these constraints and to predict the possible changes (down, neutral, up) of the activation levels of the involved players for a given experiment. Based on this formulation we developed several algorithms for detecting and removing inconsistencies between measurements and network topology. Demonstrated by EGFR/ErbB signaling in hepatocytes, our approach delivers direct conclusions on edges that are likely inactive or missing relative to canonical pathway maps. Such information drives the further elucidation of signaling network topologies under normal and pathological phenotypes.

This is a *PLOS Computational Biology* Methods article.

## Introduction

Recent advancements in high-throughput phosphoproteomic technologies have led to the generation of large datasets, capturing the cell's response to factors of its biochemical micro-environment [1], [2]. However, interpreting the increasing amounts of available data in such a manner that biologically relevant insights can be drawn for the interrogated system is far from trivial. To this end, signaling data are often examined in conjunction with network models that represent our current knowledge of the causality of cellular signal flows (as stored, for example, in online pathway databases [3]–[5]). Finding, in a rigorous fashion, causal explanations for experimental data in the context of a given network topology is one of the key challenges for systems biology of cellular signaling.

Significant work has been published on this front attempting to identify inconsistencies between measured data and signaling topologies [6]–[16]. Some methods also facilitate an optimization of the network structure to identify the wiring diagram that can best fit the data at hand [6], [7], [15]. However, before such an analysis can be conducted one has to choose an appropriate modeling formalism. Common approaches used for modeling signal transduction networks are based on graphs [12], [13], [17], [18], Bayesian networks [15], some form of logical modeling including Boolean or constrained fuzzy logic [17], [19], [20], hybrid intelligent systems [18], [19], [21]–[23], or ordinary differential equations (ODEs) [24]–[26].

Deciding on the mathematical formalism to be used for representing and modeling signal transduction networks is often not trivial and depends on many factors such as the amount and type of available data, the quality of prior knowledge, whether transient or steady-state behavior needs to be addressed, the biological questions that are to be answered, the computational efforts and so forth. For example, ODE modeling or constrained fuzzy logic are closer to the actual mechanics of signal transduction than Boolean logic as they support continuous values for the activation states of signaling species, but at the cost of numerous free parameters. These parameters must be known (in addition to the actual (initial) network structure) or estimated from experimental data. A large number of parameters in the model often gives rise to identifiability problems whose resolution requires extensive and elaborate training datasets.

**Figure 1:**
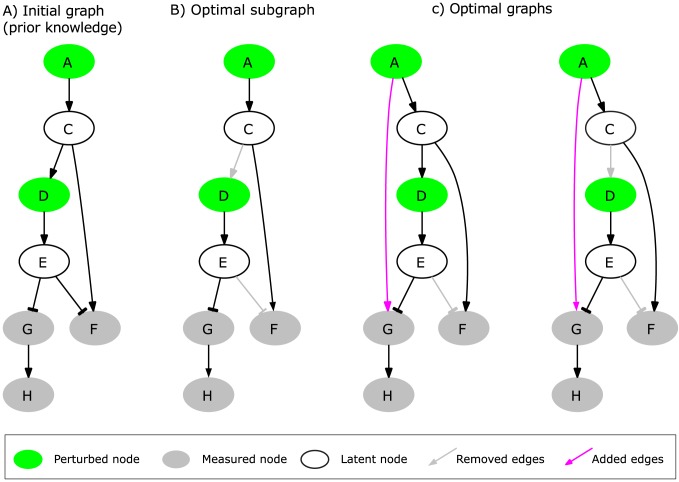
A simple example network used for illustration purposes. The interaction graph consists of 7 nodes and 7 edges. The green nodes 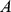 and 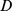 can be perturbed externally; the grey nodes 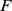, 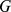 and 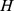 are the readouts of the network whose activation state is measured in the experiments; the white nodes 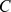 and 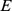 are latent nodes which are neither perturbed nor measured (see scenarios in Table 1). (A) The initial topology of the interaction graph representing the prior knowledge. This graph produces a total fitting error of 5 over the three scenarios in Table 1. (B) The (unique) optimal subgraph of (A) minimizing the total fitting error on the experimental scenarios to 2 (see Table 1). (C) Two optimal graphs obtained from (A) by applying OPT_GRAPH: by adding edge 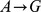 and either (left) removing 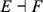 *or* (right) removing 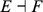 and 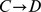, the fitting error is eradicated completely and becomes 0 (cf. Table 1).

Graph models are probably the simplest models of signaling networks one can think of. In particular, *signed directed graphs* (also called interaction graphs, dependency graphs, or influence graphs), where each edge indicates either a positive or a negative effect of one node upon another, have frequently been used to investigate basic functional properties of biological networks with signal or information flows. Despite their simplicity, interaction graphs (IG) capture the most important biological information and are useful to uncover fundamental network properties such as feedback and feedforward loops or global interdependencies between the involved players. The fact that each Boolean and each ODE model has an underlying IG renders the analysis of IG directly relevant also for other modeling formalisms. A famous example is the fact that a system (in an ODE or Boolean model representation) exhibiting bistability must contain a positive feedback loop in its underlying network structure [27], [28]. Properties that are uniquely identifiable from a given IG immediately hold for all ODE and Boolean models that have this IG as underlying wiring diagram, whereas the opposite direction does not hold. For example, in Figure 1A we see that there is (exactly) one path in the IG leading from node 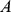 to node 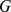 and that this path is negative. We can therefore uniquely conclude from the IG that, in any Boolean or ODE model derived from it, a perturbation in 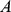 cannot lead to an increase in the activation level of 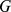. In contrast, there is a positive and a negative path from 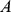 to 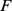, hence, nothing can be concluded from the graph alone when perturbing 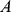. In fact, it will depend on the kinetics and parameters in an ODE model (and the logical functions in a logical model) whether the level of 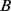 will increase, decrease, or, in the extreme case, remain constant.

The previous example shows that IG can be used to make predictions (without needing any further parameters) on the qualitative behavior of signaling and regulatory networks. These predictions can easily be compared with (qualitative trends of) experimental data, typically from stimulus-response experiments. The concept of the dependency matrix introduced in [17] is consequently based on the idea used above, namely to check—for each (ordered) pair 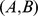 of nodes 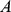 and 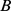
—the existence of positive and negative paths (and negative feedback loops) to make predictions on the effect of perturbations in 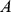. This concept has been applied, for instance, in [18] to experimental data of the epidermal growth factor (EGF) receptor signaling network. The comparisons of the predictions from the dependency matrix with the measured behavior from several combinatorial stimulations showed several inconsistencies from which some (cell-type specific) conclusions on missing or probably inactive interactions could be made. However, these conclusions were drawn by inspection only. It is therefore one goal of this study to develop methods that find, in an automatic way, corrections in the network structure improving the consistency. The dependency matrix is useful to get an overview on how a node can potentially influence the other nodes in the network; however, it may become limiting if multiple node values are measured in one experiment. Given the IG topology, state changes measured for certain nodes are, in general, not independent and therefore require stronger constraints. For example, assume there would be another node 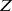 in Figure 1A that is activated by 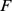 (edge 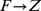). From the IG topology we know that 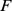 and 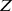 can both decrease or increase their levels if 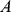 is perturbed (as correctly predicted by the dependency matrix); however, it is not possible that their new steady state levels change in different directions.

A related class of methods for detecting discrepancies between IG topology and experimental data relies on the *sign consistency rule* [11]–[13]. The key idea is that, in a steady-state shift experiment, the direction of change of the state of a node must be explainable by the direction of change of at least one of its predecessor nodes (except for the directly perturbed node(s)). For example, in Figure 1A, after a perturbation in 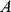, the steady-state level of 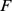 may have become larger only if 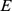 decreased its activation level (as 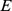 inhibits 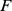) or if 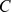 increased its level (as 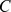 activates 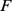). The sign consistency rule gives rise to constraints on the possible patterns of “ups and downs” of the nodes' activation levels in a given IG. These constraints can be encoded, for example, by Answer Set Programming [13]. Confronting these constraints with experimental data may then lead to the detection of topological inconsistencies, namely if no sign pattern complying with the given measurements and perturbations can be found [11]–[13].

The novel methods we will present herein are based on a similar sign consistency rule; however, they differ in a number of aspects. First, we will encode the sign constraints as an Integer Linear Programming (ILP) problem which has not been described before. This formulation gives us the opportunity to utilize the large corpus of effective algorithms developed for ILP problems. Furthermore, for the situation that multiple stimulus-response experiments are available, we will address aspects that go beyond the detection of inconsistencies from single experiments, namely to correct a given network structure such that the number of mismatches is minimized. For the structure optimization process we will consider edge removals as well as edge additions.

As starting point, we assume that we are given (i) an initial IG topology, for example, a “master topology” of a signaling pathway subsuming all reported (potential) interactions and (ii) a set of stimulus-response experiments (scenarios) in each of which some nodes were perturbed and the resulting up- or downregulation of some readout nodes was measured. The IG is a signed directed graph 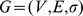, where 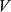 is the set of nodes (species), 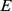 is the set of edges (interactions), and 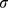 is the set of signs corresponding to edges in 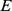 (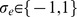, 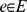). Figure 1A and the three experimental scenarios in Table 1 (defined by the columns “Perturbations” and “Measurements”) provide an illustrative example. Here, 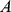 and 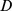 are nodes that can be perturbed; 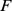, 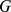 and 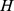 are the readout nodes for which we get measurements, and 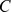 and 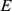 are latent nodes which are neither perturbed nor measured.

**Table 1:** Example scenarios and optimizations for the example network in Figure 1.

	Perturbations		Measurements			Initial fitting error (Fig. 1A)			MCoS	Remaining fitting error (Fig. 1B/Fig. 1C)		
	*A*	*D*	*F*	*G*	*H*	*F*	*G*	*H*		*F*	*G*	*H*
sc1	1	−1	1	1	1	0	0	0		0/0	0/0	0/0
sc2		1	0	−1	−1	1	0	0	{1→*F*}, {1→*C*}, {1→*A*}	0/0	0/0	0/0
sc3	1		1	1	1	0	2	2	{1→*G*}, {−1→*E*}, {−1→*D*}, {−1→*C*}	0/0	1/0	1/0

Rows “sc1”, “sc2”, “sc3” correspond to scenarios 1 to 3. The “Perturbations” column shows the externally imposed state of the nodes 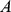 and 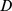 which can be −1 (downregulation), 0 (state of the node did not change), or 1 (activation level is increased). No value is given if the node was not perturbed. The “Measurements” column shows the measured change of the activation level of 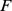, 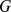 and 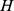 in the respective scenarios. The “Initial fitting error” column shows the total mismatch of predictions and measurements with respect to the initial topology (shown in Figure 1A). The “MCoS” (Minimal Correction Sets) column shows artificial positive (1) or negative (−1) external inputs to some nodes which would lead to a perfect fit of the data (resulting fitting error for the scenario becomes 0). The “Remaining fitting error” columns show the remaining mismatches for the optimal subgraph depicted in Figure 1B and for the two optimal graphs displayed in Figure 1C. The original network in Figure 1A has a total fitting error of 5; it is 2 for the optimal subgraph in Figure 1B and it becomes 0 in the optimal graphs in Figure 1C.

Our goal is now to analyze and improve the consistency of an IG topology with respect to a given set of experimental data. Central to all algorithms presented herein is the following definition of sign consistency.

**Definition 1** (Sign Consistency). We are given an IG and a node labeling (sign pattern) 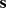 which stores for each node 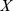 a sign 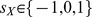. We say that 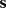 is *sign-consistent with respect to the IG* if the following conditions hold for each node 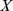:

- If 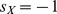: either 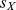 was fixed to 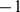 (perturbed node), or there is a predecessor node 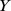 and an edge 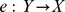 with 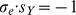.
- If 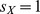: either 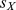 was fixed to 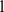 (perturbed node), or there is a predecessor node 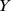 and an edge 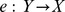 with 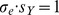.
- If 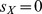: either (i) 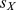 was fixed to 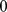, or (ii) 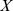 has no predecessor, or (iii) for all edges 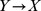 we have 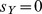, or (iv) there is an edge 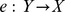 with 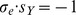 and another edge 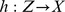 with 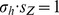.

In our setting, the signs of the external perturbations as well as the measured signs of the readout nodes can be described by a specific node labeling (which we call the *associated* labeling of the scenario). In realistic applications one usually has latent nodes which are neither perturbed nor measured, hence, the associated node labeling of an experimental scenario may contain unknown values which we denote by 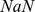. We call incomplete sign patterns *partial labelings*. A partial labeling 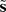 is sign-consistent if there exists a complete sign-consistent labeling 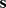 for which we have 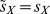 wherever 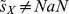. In this sense, we say that an experimental scenario is sign-consistent if its associated (partial) labeling is sign-consistent. Finally, if we have a *collection* of scenarios we say that this collection is sign-consistent with the IG if all the (partial) labelings associated with the scenarios are sign-consistent.

We can now consider four fundamental problems on the consistency of experimental scenarios with respect to a given IG:

### (1) SCEN_FIT

Given a single experimental scenario, we fix the states of the perturbed nodes (according to the experimental interventions) and then search for a sign-consistent node labeling having a minimal mismatch with the given measurements. In the ideal case, where the associated labeling of the experimental scenario is sign-consistent, the *fitting error* will be 0. The fitting error is defined as the absolute difference 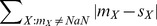 between the measurements 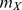 and the optimal sign pattern 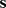.

From Figure 1A/Table 1, we see that scenario 1 is sign-consistent: 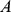 was externally increased and 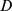 decreased, and with 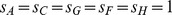 and 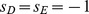, we obtain a sign-consistent labeling giving us a possible explanation for the measurements. In contrast, scenario 2 is not consistent with the IG topology: if 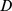 is increased externally (no perturbation in 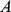), then we expect to see a decrease in 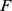, 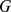 and 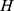 which is not seen in 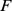 (unchanged). The minimal resulting fitting error for an optimal sign pattern is thus 1. Generally, an error of 1 or 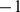 occurs if a change was expected/not expected, but was not seen/was seen in the experiments. For scenario 3, the predictions are even worse: increase in 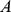 (no perturbation in 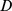 which thus depends on 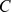) should lead to down-regulation of 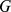 and 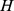, but an increase is measured for both. We thus get an absolute error of 2 for each of the two predictions. The fitting error of a sign-consistent node labeling closest to scenario 3 can thus not be smaller than 4.

It may happen that several solutions exist explaining a given scenario equally well. For example, assume again that there was another node 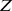 in Figure 1A that is activated by 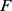 through an edge 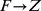. If we now measured 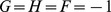 and 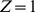 after positively perturbing 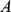 (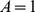), then the best scenario fit would result in an error value of 2 since 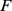 and 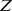 must have the same value. However, there are three optimal solutions regarding 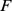 and 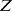, namely 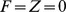, 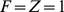, and 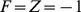, all leading to the same minimal fitting error of 2. For some applications it will be helpful to know all these optimal solutions and we will therefore also address their enumeration.

### (2) Minimal Correction Sets (MCoS)

Another optimization problem for a single scenario directly follows if a given scenario is not sign-consistent, i.e., if no sign-consistent labeling can be found that results in a fitting error of 0. We can then try to identify a minimal set of nodes whose states need to be corrected to obtain a consistent scenario. The correction of a node's state is simulated by adding an additional external input that is either 1 or 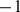. We call these sets *Minimal Correction Sets* (MCoS), the minimality property demanding that no subset of a MCoS would lead to a consistent labeling. For example, regarding scenario 3 in Table 1, there are four MCoS suggesting that there was either an external up-regulation of 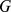 (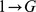), or a down-regulation in one of the nodes 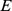, 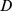, or 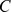, each of unknown cause. Thus, MCoS show possible places in the network that have a high probability to cause the observed inconsistencies. With the MCoS problem we identify the enumeration of MCoS of minimal size for a given scenario (a simple extension not considered herein is to enumerate all MCoS irrespective of their size).

### (3) OPT_SUBGRAPH

The first two problems focus on a single scenario; now we intend to optimize the network structure in such a way that the total fitting error over all scenarios is minimized. Initially, we allow only the removal of edges in the network, that is, we search for an optimal subgraph. As there might be several solutions to this optimization problem, we consider the following sub-problems: computation of any/of the sparsest/of the largest sub-network of the initial IG minimizing the mismatches. In addition, we may also be interested in an enumeration of all sub-networks minimizing the number of inconsistencies between IG topology and data. As an example, Figure 1B shows the unique optimal subgraph of the original IG in Figure 1A minimizing the fitting error over all three scenarios in Table 1. This solution reduces the total fitting error from 5 to 2 (and there is no solution that could reduce it further).

### (4) OPT_GRAPH

The removal of certain edges may significantly improve the agreement between measurements and network topology, but some fitting errors can often only disappear if we have additionally the opportunity to add new interactions. This fourth optimization problem, therefore, intends to minimize the fitting error by allowing edge removals *and* insertions in parallel. Obviously, the fit cannot be worse than the one obtained by problem (3). For smaller networks, a full enumeration of all optimal solutions might be possible. However, as the insertion of new interactions increases the solution space dramatically in large networks, we may consider a *greedy* strategy which determines, in each iteration, the optimal edge whose inclusion (in combination with the pruning step (3)) decreases the fitting error the most. One may then add this edge permanently and repeat the algorithm described above until no further significant improvement can be obtained by inserting a new edge.

Figure 1C shows a result of this optimization step in our example: the edge 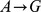 is identified as missing edge which, in combination with a pruning step, completely eradicates the original fitting errors in all scenarios. The resulting network is thus fully consistent with the entire set of experimental data. In this example, nine other edges can be identified whose addition, in combination with a pruning step by OPT_SUBGRAPH, lead to a fitting error of 0. Furthermore, for each added edge, the OPT_SUBGRAPH problem that is called after adding the edge might return several optimal solutions. Figure 1C shows the two existing optimal solutions (with a fitting error of 0) that are derived after adding edge 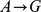.

The present paper is organized as follows: the Methods section details how sign consistency and the four basic optimization problems can be encoded as Integer Linear Programming problems. The Methods section thus contains the main theoretical achievements of our work. Readers not interested in the mathematical details may skip this part and directly continue with the Results section. In the latter we employ our proposed methodology to identify the EGFR/ErbB signaling topology active in primary hepatocytes [18] by using prior knowledge on network topology and data from combinatorial stimulus-response experiments. This study reveals interesting biological insights and demonstrates that the introduced framework provides a highly flexible and powerful approach for exploring and training wiring diagrams of signaling networks based on large sets of experimental data. We also provide results from benchmarks of our algorithms and discuss the scalability of the presented method.

## Methods

### Basic definitions and ILP formulation of sign consistency

As described in the Introduction section, we assume that we are given an interaction graph (signed digraph) 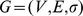 capturing our prior knowledge on the signaling topology and, additionally, a set of experimental scenarios each consisting of a specific set of perturbed nodes and a set of measurements. The edges (also called interactions) are indexed by 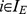, 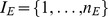, 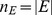, the nodes by 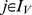, 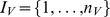, 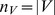, and the scenarios by 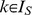, 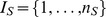. The experimental scenarios are specified by two matrices: (i) the 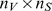 perturbation matrix 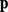 with 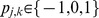 storing the (enforced) state of node 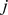 in scenario 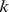 through external perturbation, and (ii) the 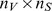 measurement matrix 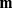 with 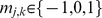 storing the measured change of the (steady) state level of node 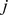 in scenario 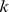. Perturbation and measurement values thus indicate enforced/measured upregulation (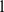), downregulation (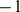), or unchanged state (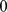). Usually, only a small subset of nodes is perturbed, and only a subset of nodes can be measured; unperturbed and non-measured states are therefore marked by 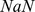 in the matrices 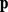 and 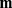, respectively.

In what follows we translate sign-consistency of a node labeling (according to Definition 1) into equality and inequality constraints of an Integer Linear Programming (ILP) problem. In this formulation, the predicted state of a node 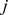 in experiment 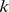 will be represented by an integer variable 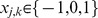. Again, 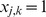 encodes upregulation and 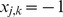 downregulation of node 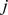 in scenario 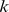, whereas 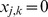 indicates that the activation level of 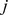 remained unchanged.

The 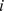-th signaling edge is defined as 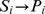, where 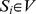 is the start node and 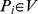 the end node of edge 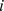. Furthermore, the sign of edge 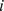 is denoted by 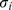.

We introduce the binary variables 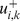 and 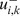 to represent the potential of edge 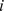 to up- or downregulate its end node 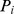 in experiment 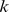. Edge 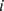 with start node 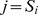 has the potential of upregulating its target node 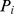 in experiment 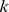 (i.e., 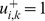) if and only if 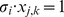. In any other case we have 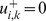. Accordingly, edge 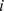 with start node 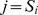 has the potential of downregulating its target 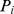 in experiment 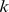 (i.e., 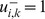) if and only if 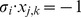. In any other case 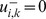. Thus, with 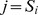,
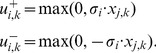
(1)As the max operator is not linear (required for an ILP), we introduce the binary variables 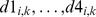 to linearize (1) in the following way:
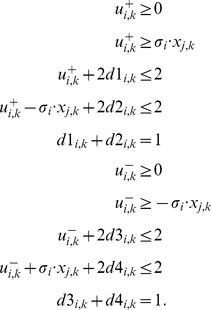
(2)Finally, the two binary variables 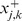 and 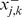 are introduced to represent the potential for node 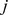 of being up- or downregulated depending on the activity of its upstream edges. Node 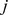 has the potential of being upregulated (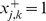) if and only if an edge 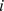 exists such that 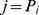 and 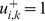, and node 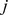 has the potential of being downregulated (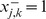) if and only if 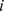 exists such that 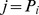 and 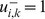. Thus,
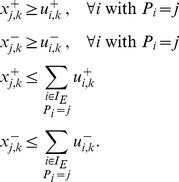
(3)The state 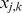 of node 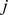 in scenario 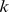 is constrained by the values of 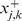 and 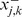 according to the definition of sign-consistency (see Definition 1): (i) Node 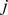 may be upregulated (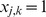) if it has the potential of being upregulated (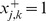). (ii) Node 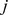 may be downregulated (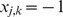) if it has the potential of being downregulated (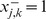). (iii) Node 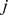 may stay unchanged (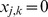) if it has the potential of being both up- and downregulated (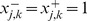) or neither of the above (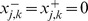). These rules are encoded in inequalities as follows:
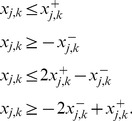
(4)The equations and inequalities derived in this subsection describe sign-consistent node labelings and provide the frame within which we can now address the four basic optimization problems posed in the Introduction section.

### SCEN_FIT

The goal of SCEN_FIT is to identify, for a given scenario 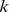, a sign-consistent vertex labeling that is closest to the measurements of this scenario. We first have to constrain the values of the perturbed nodes in scenario 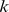:
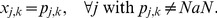
(5)Realistic perturbations typically affect either input nodes (e.g., ligands) or internal nodes in the case where a specific inhibitor was added or where a constitutive activation or a knock-in/knock-out is introduced. The state of the perturbed nodes are thus fixed to the enforced value and the constraints (4) are omitted for these nodes to preserve the consistency of the formulation.

We now search for a sign-consistent labeling 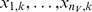 (fulfilling thus constraints (2)–(4) of the previous subsection) that minimizes the measurement-prediction-mismatch. The following objective function is used accordingly:
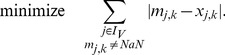
(6)The summation of mismatches in equation (6) is thus done over all nodes for which measurements exist. By introducing 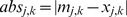, 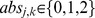, the lower bound for the absolute value of the mismatch above is formulated as follows (an upper bound needs not to be defined because the objective function (6) will automatically take the smallest possible value):
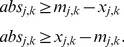
(7)The resulting states 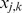 for scenario 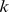 represent an optimal solution as desired for SCEN_FIT.

As discussed in the Introduction section, we also consider the enumeration of *all* optimal SCEN_FIT solutions for a given scenario. To this end, we solve the ILP repeatedly and after each run we exclude previously found solutions by adding the following constraints for each previous solution 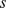:
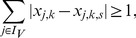
(8)where 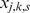 represent the value of 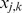 in solution 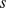. Since constraint (8) is again non-linear because of the absolute value, it is reformulated in the following manner:
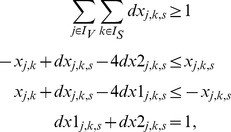
(9)with the auxiliary variables 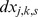 (integer) and 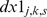 and 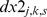 (binary). We may then compute a new sign-consistent labeling of the nodes by optimizing again objective function (6). To ensure that only solutions with minimum fitting error are found, we replace, after the first iteration, the objective function in (6) by forcing instead the algorithm to find solutions with the same minimum fitting error as in the first run:
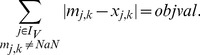
(10)Here, 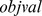 is the optimal (minimal) value of the objective function (6) found in the first run of the algorithm. The resulting problem becomes thus a simple search for a feasible solution and is repeated until no further solution can be found.

### Minimal Correction Sets

#### Computing a single Minimal Correction Set

Next, we address the identification of a Minimal Correction Set (MCoS) for a sign-inconsistent scenario 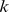 (where the fitting error in equation (6) after optimization is greater than zero). An MCoS indicates possible causes of discrepancies between measured data and assumed IG topology. As described in the Introduction section, MCoS correspond to artificial perturbations of certain nodes which render the measurements from a given inconsistent scenario consistent with the network topology. Let a new set of binary variables 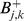 and 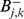 denote these artificial perturbations. The state 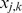 of node 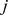 can be enforced to 1 by adding a positive input, 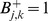. Accordingly, 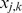 can be enforced to 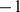 by adding a negative input, 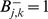. To enforce the state of 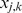 to 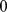, either a positive (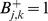) or a negative (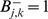) input might be required. To account for these artificial perturbations, we modify the constraints (4) in the following manner:
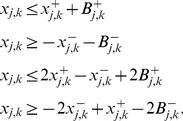
(11)Having introduced the correction terms 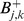 and 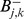, we set as an extra constraint the perfect fit for all measured nodes (which is now always feasible):
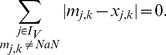
(12)The absolute value is again reformulated as described in section SCEN_FIT. As we are interested in MCoS with a *minimum* number of corrections, we use the following objective function:
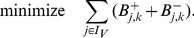
(13)


#### Enumeration of Minimal Correction Sets

In general, many MCoS of minimum size may exist; therefore, we address in this subsection the enumeration of *all* minimum MCoS. To this end, we solve the ILP repeatedly, and after each run, we exclude previously found solutions by adding the following constraint (so-called integer cuts) for each previous solution 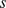:
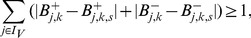
(14)where 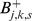 and 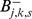 represent the value of 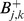 and 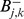 in solution 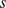. Constraint (14) can be linearized as follows:
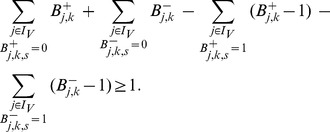
(15)We may then compute a new MCoS by optimizing again objective function (13). To focus only on MCoS with the minimum number of corrections, we replace after the first iteration the objective function (13) by forcing the algorithm to find a solution with the same minimum number of corrections:
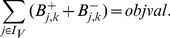
(16)Here, 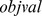 is the value of the objective function found in the first run of the algorithm. The resulting problem becomes thus a simple search for a feasible solution and is repeated until no further solution can be found.

### OPT_SUBGRAPH

#### Computing a single optimal subgraph

As stated in the Introduction section, OPT_SUBGRAPH searches for an optimal subgraph of the original topology (i.e., for a set of suitable edge removals) minimizing the total fitting error *over all* scenarios. In this subsection we describe how we can identify one particular solution to this problem before turning to the enumeration of optimal subgraphs.

The removal of edges is implemented using binary variables 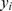. The algorithm will set 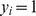 if the edge 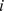 is removed by the optimization procedure to improve the fit of the data (otherwise 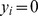). We impose again the constraints (1)–(4) for sign-consistency. The actual pruning is implemented by modifying constraints (1) as follows:
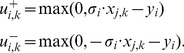
(17)The max operator is again rewritten in form of linear constraints:
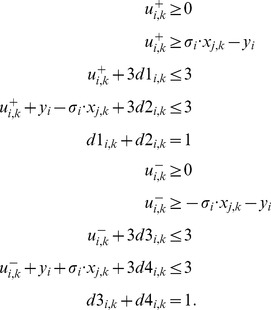
(18)We then reuse objective function (6), but now minimize the measurement-prediction mismatch *over all* scenarios:
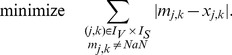
(19)


This optimization will deliver an optimal sub-network of the original IG which can best explain the data. Usually, many optimal solutions may exist yielding the same residual fitting error in Equation (19). One might then be interested to focus on particular solutions, for example, on those containing the minimal/maximal number of edges in the remaining subgraph. For this purpose, we may replace (19) by
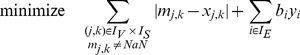
(20)(the absolute value is again reformulated in form of linear constraints). The constant 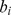 is defined as follows: in order to arrive at a solution with minimal error between predicted and measured values, the absolute value 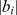 needs to be less than 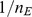. Furthermore, constants 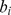 assume negative values (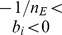) for obtaining a minimum subgraph and positive values (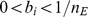) for obtaining a maximum subgraph.

Another way to deal with non-unique solutions is to enumerate all of them which we address next.

#### Enumeration of optimal subgraphs

To identify all optimal subgraphs minimizing the inconsistencies between IG topology and measurements of all scenarios, we solve the ILP repeatedly and after each run we exclude previous solutions 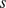 by adding the following constraints:
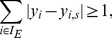
(21)where 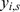 represents the value of 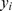 in solution 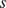. Constraint (21) is reformulated in linear form as follows:
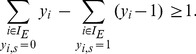
(22)


Moreover, after the first run we replace the objective function in (19) by enforcing the algorithm to obtain the same, optimal, goodness of fit as in the first run:
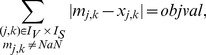
(23)where 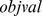 is the value of the objective function (19) after the first run of the algorithm. In the same way we may also consider the enumeration of minimum and maximum subgraphs; we then have to fix (20) to its optimal value instead of considering (19).

### OPT_GRAPH

As motivated in the Introduction section, optimizing the IG topology by edge removals may eliminate some, but often not all mismatches. One reason could be that some real effects cannot be transduced in the model due to missing edges. We therefore propose an algorithm suggesting de-novo interactions whose addition would minimize the fitting error. As the possibility to insert new interactions increases the solution space dramatically in large networks, we consider the following greedy strategy: for each interaction not contained yet in the IG, we temporarily insert this edge and determine the resulting optimal solution for the fitting error by applying the OPT_SUBGRAPH algorithm introduced above. The single interaction that reduces the fitting error the most is picked by the greedy algorithm and permanently inserted in the IG. This process is repeated until no further edge exists that could improve the goodness of fit to the data significantly (significance can be quantified by a certain threshold). Importantly, at the beginning of each iteration, a list of eligible edges is computed consisting only of those edges that do not form a positive cycle (see below).

### Positive cycles and steady-state assumption

(Feedback) cycles often hamper the analysis of causality and many network inference techniques therefore exclude cycles from the network or assume that no cycles exist (see, e.g., [7], [15]). In contrast to many other approaches, our method can readily deal with negative cycles without any problems. However, positive cycles may become problematic as they can provide explanations for state changes without any external perturbation. A simple example for such “self-explaining” state changes is the following network: 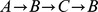 (all edges are positive). Node 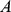 would normally serve as an input. However, assuming that 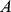 has not changed, a measured up-regulation of 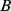 would be explainable by the sign-consistent labeling (0,1,1), that is, 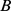 activates 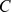 which then activates 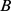 again. Although such a shift without external perturbations could indeed happen in realistic systems (due to fluctuations in bistable systems), we recommend that the initial IG should not contain a positive feedback (otherwise, many observations might become sign-consistent just through the existence of positive cycles). This is also the reason why a new candidate edge can only be added to the network if it does not give rise to a new positive cycle (see previous subsection). In many applications, this requirement is not a real limitation, in particular when describing early events in signaling networks.

We also restate another assumption for the analysis followed herein, namely that the system moves from one steady state to another upon imposing the perturbations (see also [11]; similar assumptions are also required in other studies, e.g., [7], [29]). However, this does not necessarily mean that we have to wait until the system has reached its new steady state completely; instead, we can take the measurements if we can assume that the *signs* of the state variations will not change anymore. It will therefore be important to determine a suitable time point where all relevant state changes induced by the perturbation have become visible in the measurements. For example, if measurements are taken too early, a signal has possibly not yet been propagated to all downstream nodes at the bottom of the network resulting in inconsistencies with the predictions made from the IG.

### Model compression

**Figure 2:**
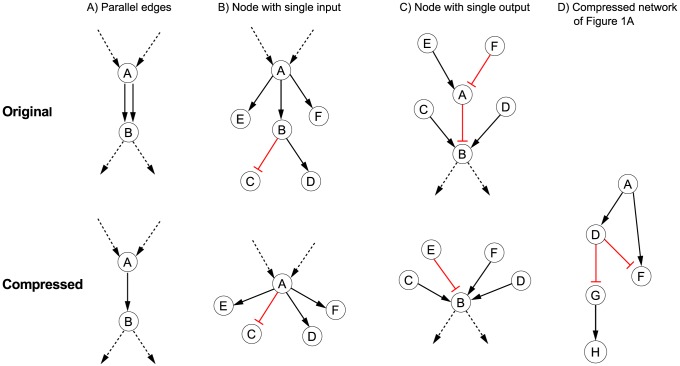
Basic network compression rules. (A) Parallel edges. (B) Nodes with single input. (C) Nodes with single output. (D) Shown is the compressed version of the network in Figure 1A after applying the compression rules. For further explanations see main text.

In the previous sections we presented several ILP formulations related to detecting and resolving inconsistencies between IG and experimental data. As long as one searches for a single (optimal) solution it is likely that a solution will be found even in very large networks due to an evolved library of effective ILP algorithms (see also benchmarks discussed in the Results section). However, the related enumeration approaches may quickly become intractable, at least if one aims at an exhaustive enumeration. In those cases one may stop the calculation if no new solution is found within a given time interval. Another useful strategy is to use (loss-free) network compression techniques by which (compressed) solutions can be calculated from a smaller network and then subsequently decompressed to solutions of the full network. Other advantages of network compression are that differences between the original and the compressed network structure may indicate non-identifiabilities in the original network and that obtained optimal solutions can be represented in a condensed manner (not explicitly displaying all combinatorial solutions existing due to non-uniqueness). We use four simple compression rules (illustrated in Figure 2) in an iterative manner which, as shown in the EGF scenario below, may reduce the network size considerably so that enumeration of solutions in large networks become possible (some but not all rules are identical to those used in [7]). Compressing the network is particularly useful for enumerating solutions for OPT_GRAPH and OPT_SUBGRAPH.

**Rule 1** (removal of non-controllable and non-observable nodes): *Non-controllable* nodes (which cannot be affected by any of the perturbed nodes in any scenario) and *non-observable* nodes (which do not influence any measured (readout) node in any scenario) define non-identifiable parts of the network. Therefore, these nodes as well as all edges they are connected to can be removed. Non-observable and non-controllable nodes can easily be identified by shortest path algorithms (cf. [7]).
**Rule 2** (removal of parallel edges): If there are two parallel edges of the same sign, we may safely remove one of them (Figure 2A).
**Rule 3** (absorbing a node with a single input edge): If a latent node (neither measured nor perturbed in any of the experimental scenarios) has only one single incoming edge, then we can remove this node (together with the incoming edge) and reconnect all the outgoing edges of this node to its only predecessor node (under consideration of edge signs; see example in Figure 2B).
**Rule 4** (absorbing a node with a single output edge): If a latent node has only one single outgoing edge, then we can remove this node (together with the outgoing edge) and reconnect all its incoming edges to its only successor node (under consideration of edge signs; see example in Figure 2C).

Rule 1 is performed once at the beginning, whereas rules 2–4 are iteratively used until no further rule can be applied (note that new parallel edges may arise after applying rules 3 or 4). The compressed version of the example network in Figure 1A is shown in Figure 2D).

By keeping track of the made compression steps it is, in principle, possible to decompress solutions found by the described optimization algorithms in the compressed network. However, as mentioned above, it is often useful to discuss the obtained solutions directly in the compressed network, thereby avoiding the interpretation of a typically much larger number of decompressed solutions arising due to non-uniqueness. For example, instead of listing all possible (parallel) pathway combinations connecting 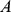 with 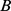, one might conclude that “at least one pathway between A and B must exist” which can easier be represented in a compressed network.

### Implementation: *SigNetTrainer*

The ILP formulations presented in the previous sections were implemented in the new software *SigNetTrainer*. The toolbox is available in two versions, the first is written in C and uses routines from the ILP solver GUROBI (http://www.gurobi.com), whereas the second version is implemented in MATLAB and uses the IBM ILOG CPLEX Optimizer (for which free academic versions can be obtained via http://www-03.ibm.com/ibm/university/academic/pub/page/membership) as ILP solver. Thus, *SigNetTrainer* benefits from state-of-the-art-solvers for ILP problems which use a number of methodologies to deal with large-scale problems. For a more general introduction to ILP algorithms we refer to [30].

*SigNetTrainer* is easy to use; the user has to provide three files to define network training problems: (i) the network topology in.sif format (also used by Cytoscape http://www.cytoscape.org), (ii) an ASCII file describing the experimental scenarios (i.e., the imposed state changes), and (iii) an ASCII file containing the experimentally measured state changes for each scenario. The user may then call different functions implementing the optimization routines as described herein. Source code and manual of both versions of *SigNetTrainer* are available on the following website:

http://www.mpi-magdeburg.mpg.de/projects/cna/etcdownloads.html.

Preprocessing routines, in particular the network compression algorithm, were implemented as MATLAB functions and are also part of the package. The manual of *SigNetTrainer* is provided in the Supporting Information (Text S1).

## Results

### EGFR/ErbB signaling in hepatocytes

In order to demonstrate the performance of the proposed approach in a realistic situation, we apply it to a recently published network topology of EGFR/ErbB signaling [18] with the aim to identify topological particularities of this important signaling pathway in hepatocytes. The network was built within the logical modeling framework introduced in [17] and describes signal transduction downstream of the members of the EGF receptor family, ErbB1–4. Network reconstruction was based on signaling reactions reported in literature and databases. As the included reactions have been observed in a variety of cell types and tissues, the model must be seen as a “master network” and it is likely that not all of the included interactions are functional in primary human hepatocytes considered herein. In [18], qualitative predictions derived both from the logical model and its underlying interaction graph were compared with a dataset (a subset of the phosphoproteomic data published in [2]) consisting of combinatorial treatments of primary human hepatocytes with/without TGF*α* and specific molecular inhibitors (see Figure S1). Note that the measurements were taken at an optimal time point such that the perturbation-induced changes in the phosphorylation level of the proteins are well-reflected by the measurements [2]. The interaction graph-based data analysis in [18] made use of the dependency matrix of the network (see Introduction section): for pairs of experiments (e.g., Exp. 1: stimuli 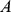, inhibitor 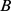, Exp. 2: stimuli 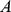, no inhibitor) it was checked whether the ratio of the measured responses (e.g., Exp. 1/Exp. 2, showing the effect of inhibitor 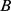) is consistent with the causal dependencies in the network topology (e.g., if 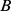 has a positive/negative/no influence on a readout 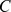, inhibiting 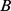 should lead to decreased/increased/unchanged 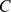). Resulting from this analysis, changes in the network structure were proposed that would improve the agreement between experimental data and model predictions. These changes were derived solely by inspection; the ILP approach presented herein can be seen as a step forward as it adapts the model structure to the experimental data in an automatic way and searches systematically for all possible solutions resolving discrepancies between model and data.

### Preprocessing

**Figure 3:**
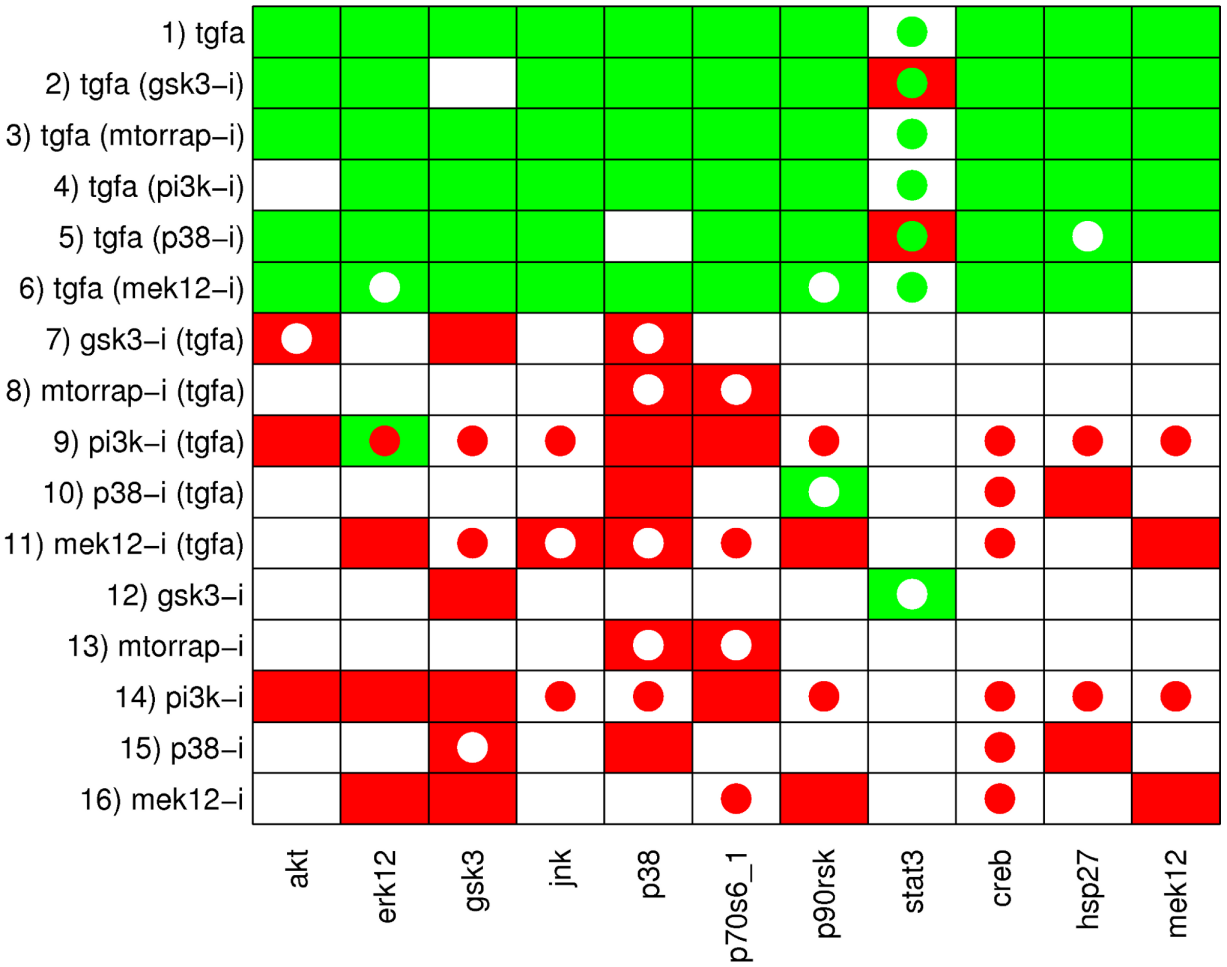
Discretized measurements of the 16 considered experimental scenarios and the resulting SCEN_FIT solutions computed from the EGFR/ErbB graph model. Each row corresponds to one experimental scenario, each column contains the measured state changes of the readout species. The discretized measurements are mapped to the fill color of the respective fields: if a node is upregulated in the respective scenario, the corresponding field is filled green, if it is downregulated, the field is filled red, and if it shows no significant change, it is filled white. Accordingly, the color of the added circles shows the sign of the node in the closest sign-consistent node labeling derived by SCEN_FIT: green circles correspond to sign 1, red circles to sign −1 and white circles to sign 0. Note that circles only appear if the measurement is not in accordance with the respective state in the sign-consistent labeling.

Before applying the ILP formulation, both the phosphoproteomic data (Figure S1) and the EGFR/ErbB signaling network topology used in [18] had to be preprocessed. The phosphoproteomic data were originally obtained via xMAP technology which measures fluorescent units [2]. The dynamic range of the measured signals depends on the antibody pair used for detection. For example, the signal for JNK ranges from 100 units to 500 units, while MEK1/2 ranges up to 25000 units (Figure S1). Variations such as these do not necessarily reflect that JNK is less activated than MEK1/2, but may be attributed to protein abundance or assay calibration issues. Furthermore, the proposed formulation requires a qualitative view of signal transduction, supporting only three discrete states indicating the variation of the activation state of signaling nodes when changing external inputs or adding inhibitors (“−1” for downregulated, “0” for unchanged, and “1” for upregulated). Thus, the raw data need to be discretized before it can be used in the ILP formulation. To this end, the methodology introduced by Samaga et al. in [18] is adopted: the ratios of all experiments that differ only by a single perturbation (ligand or inhibitor treatment) are evaluated and the respective measurement is considered to be (i) upregulated if the fold-increase of the signal (with versus without perturbation) is above 1.5, (ii) downregulated if the fold-decrease of the signal (with versus without perturbation) is below 0.66 and (iii) unchanged otherwise. The dataset analyzed in [18] contains measurements with JNK inhibitor showing an effect of the inhibitor on many of the measured signals. As these inhibitions are likely to be off-target effects [2], we decided to exclude the JNK inhibitor data for our analysis. The complete set of discretized data can be seen in Figure 3.

**Figure 4:**
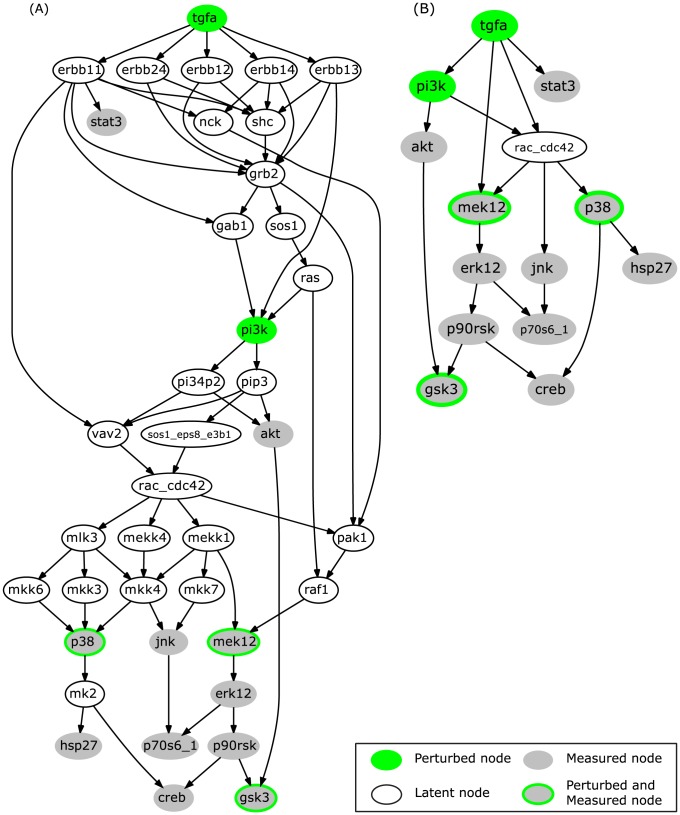
Interaction graph model of the EGFR/ErbB signaling network. (A) The full network adopted from [18] after removal of non-observable and non-controllable nodes. All edges are activating edges (having positive signs). (B) The compressed model obtained after applying the compression rules to (A).

Regarding the EGFR/ErbB network model, the original interaction graph used by Samaga et al. [18] was adopted but non-observable and non-controllable nodes were removed (see [7] and Rule 1 of the model compression described in the Methods section; the full compression will be applied in a later step). The resulting graph is shown in Figure 4A.

### Applying SCEN_FIT and Minimal Correction Sets

Figure 3 depicts the discretized measurements and, for each scenario, the corresponding SCEN_FIT solution. Recall that the SCEN_FIT algorithm determines, for a given scenario, a sign-consistent node labeling that is closest to the measurements and can thus best explain how the EGFR network topology in Figure 4A induces the measured node changes for the respective scenario. Deviations between the determined optimal sign pattern and the measured state changes (as indicated in Figure 3) uncover inconsistencies between network structure and observed behavior. For example, scenario 1 reflects the influence of the ligand TGF*α*, that is, TGF*α* is the perturbed node and its state is fixed to 1. As depicted in Figure 3, the SCEN_FIT solution for this scenario shows a fitting error of 1: in the optimal sign-consistent node labeling, all measured nodes have sign 1 as they are connected to TGF*α* by positive paths only. This is in accordance with the measured state of all nodes except STAT3: the latter shows no significant change in response to TGF*α* inducing thus a fitting error. Scenarios 2–6 reflect the influence of TGF*α* in presence of different inhibitors. We assume that an inhibitor completely blocks the signal flow through the inhibited species and thus define these scenarios by fixing the state of TGF*α* to 1 and of the inhibited node to 0. The remaining scenarios reflect the influence of the inhibitors in presence (scenarios 7–11) and absence (scenarios 12–16) of TGF*α*. In each of these scenarios the perturbed node is the respective inhibitor and its state is fixed to −1. Importantly, by using the enumeration algorithm for SCEN_FIT we could prove that, for each scenario, the found solution for the optimal fit is unique, hence, no other optimal solutions need to be considered. We also assessed the sensitivity of the SCEN_FIT results with respect to the chosen thresholds for data discretization and found a fairly robust behavior for a relatively large range of the threshold parameters (see Figure S2 and Text S2).

Figure 3 shows that there are several inconsistencies between experimental data and the SCEN_FIT solutions derived from the initial network topology. In order to understand where these inconsistencies are induced in the network, we address the identification of minimal correction sets (MCoS). We recall that MCoS are minimum sets of (artificially) enforced changes of node states (e.g., from up- to downregulated) which make an inconsistent scenario consistent. Exemplarily, we focus on scenario 14 of Figure 3 (where PI3K-i is added without presence of TGF*α*) whose SCEN_FIT solution produced a total error value of 6.

As shown in Table 2, five MCoS are identified, each containing three corrections (virtual perturbations) rendering the experimental scenario 14 sign-consistent. Common trend in all MCoS is to remove the downregulating effect of PI3K on signals downstream of Rac_Cdc42 by setting Rac_Cdc42 to unchanged (0) or one of the nodes SOS1_Eps8_E3b1, Vav2, PI(3,4)P2 or PIP3 to upregulated (1). Introducing this change, the states of p38, JNK, MEK1/2, Hsp27, CREB and p90RSK are now in accordance with the measurements (i.e., they show now response upon adding PI3K inhibitor). However, by this modification, the states of ERK1/2 and p70S6_1 would change their predicted level from “downregulated” to “unchanged” which is not in agreement with the measured state. This is corrected in all MCoS by setting ERK1/2 to −1. Again, this correction implies an undesired effect, namely changing p90RSK from 0 to −1, which is countered by assigning p90RSK the value 0 in all MCoS. Clearly, three required corrections indicate that the observed behavior for this scenario is not well-reflected by the network topology. It would therefore be useful to consider all scenarios at the same time to detect common points of errors produced in all or many scenarios.

**Table 2:** MCoS for scenario 14 in Figure 3.

	MCoS 1			MCoS 2			MCoS 3			MCoS 4			MCoS 5		
Node id	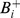	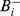	Val	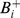	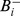	Val	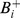	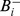	Val	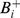	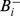	Val	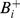	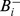	Val
rac_cdc42	1		**0**												
p90rsk	1		**0**	1		**0**	1		**0**	1		**0**	1		**0**
erk12		1	**−1**		1	**−1**		1	**−1**		1	**−1**		1	**−1**
sos1_eps8_e3b1				1		**1**									
vav2							1		**1**						
pi34p2										1		**1**			
pip3													1		**1**

Five MCoS are identified for the EGFR network model (Figure 4) with respect to scenario 14 in Figure 3. Each MCoS would lead to a perfect fit for this scenario and all five MCoS contain three nodes to be enforced to a certain value. Nodes p90rsk and erk12 are common in all MCoS. Nodes rac_cdc42, sos1_eps8_e3b1, vav2, pi34p2 and pip3 are perturbed respectively in MCoS 1–5. In columns MCoS 1–5, three sub-columns are shown: sub-column “Val” shows the corrected state of the node (the actual MCoS), the entry 1 in sub-column “
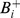
” indicates that a positive input edge is added to the node in order to alter its state, and the entry 1 in sub-column “
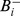
” indicates that a negative input edge is added to the node (see Methods section).

### Applying OPT_SUBGRAPH

We use the OPT_SUBGRAPH algorithm to find—by appropriate edge removals—an optimal subgraph of the EGFR network structure which minimizes the fitting errors over all experimental scenarios.

To be able to make meaningful conclusions, we need to find all optimal solutions. However, enumerating all solutions for OPT_SUBGRAPH in the full model structure becomes quickly intractable as the highly branched network structure (e.g., various feedforward routes running over different combinations of ErbB dimers and adapter proteins connect TGF*α* with PI3K) leads to an immense number of different optimal solutions. Therefore, we compress the model structure as described in section “Model compression” before searching for optimal subgraphs. As can be seen in Figure 4B, the model structure can be compressed substantially from 39 nodes and 67 edges to 14 nodes and 18 edges. Strikingly, Rac_Cdc42 remains as the only latent node in the compressed structure. The compressed IG reflects the essential dependencies in the original network structure that can be addressed by the given set of perturbed/measured nodes. For example, parallel signaling paths leading from a perturbed node to a measured node without passing any other measured/perturbed node cannot be distinguished in the analysis performed herein and are therefore condensed to one single edge in the compressed graph.

**Figure 5:**
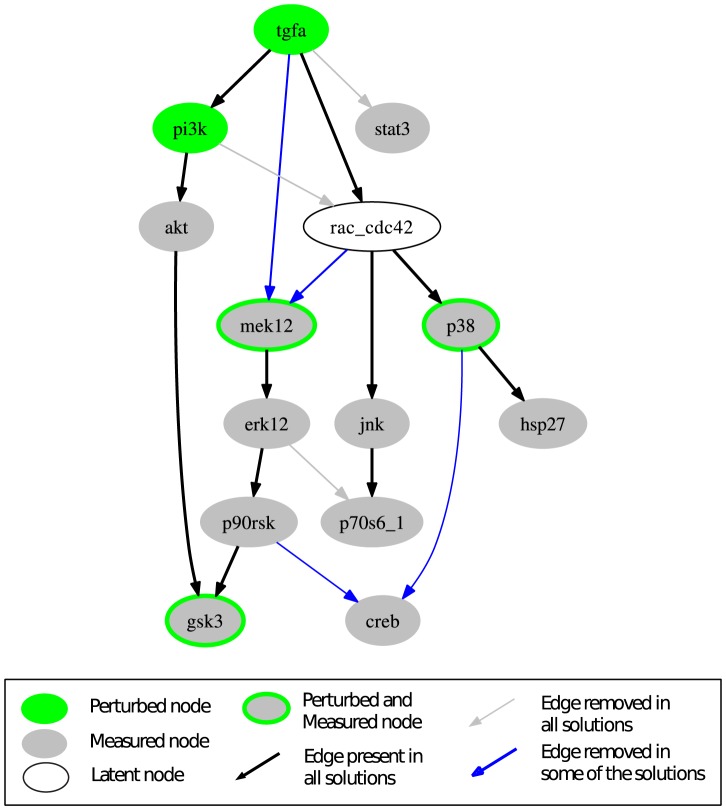
Combined view of all optimal model structures derived from the compressed EGFR/ErbB model by applying the OPT_SUBGRAPH procedure with enumeration.

**Figure 6:**
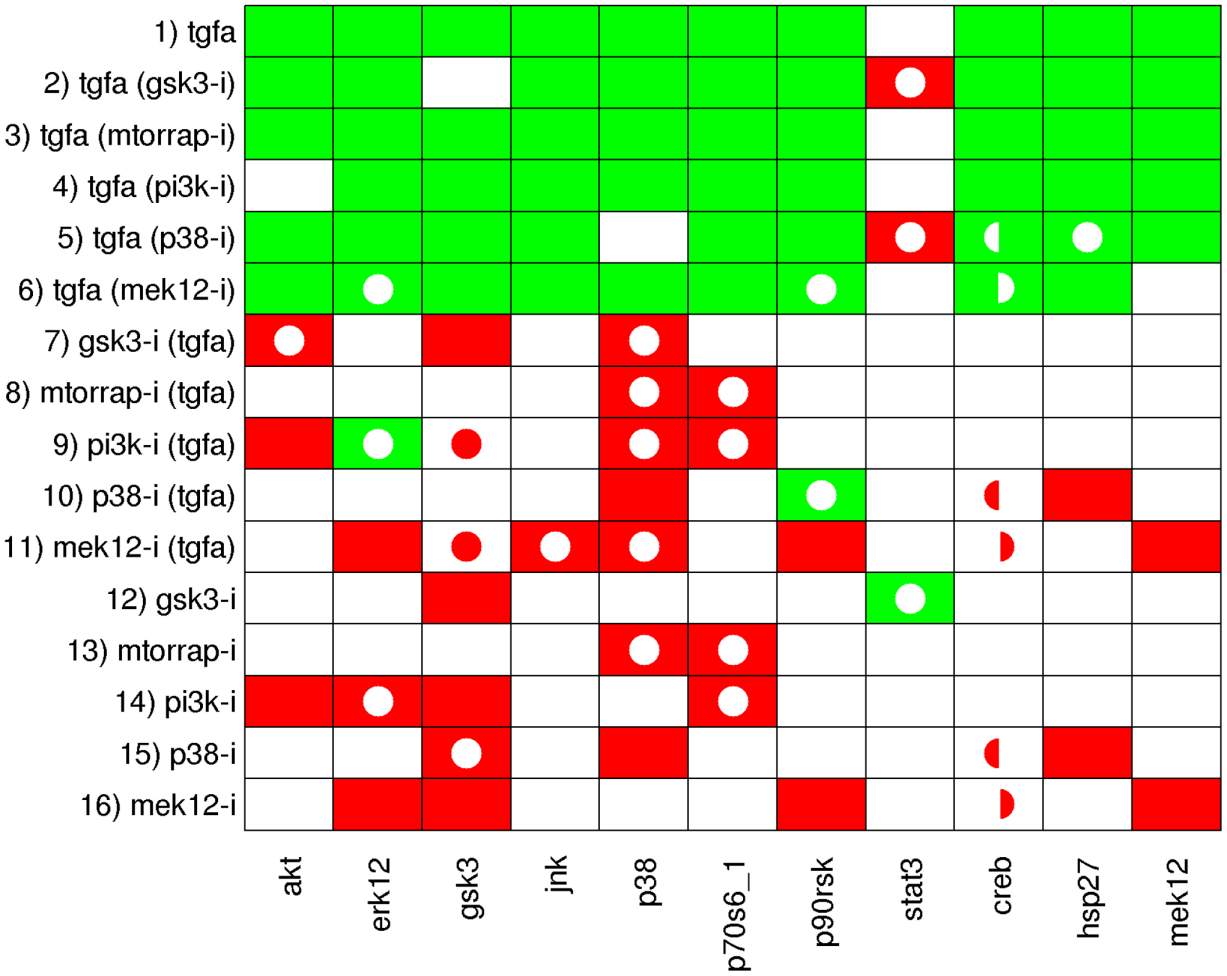
Discretized data and the (two) SCEN_FIT solutions that result from the optimal subgraphs given in Figure 5. The color coding is the same as in Figure 3. All six optimal subgraphs contained in Figure 5 give rise to the same SCEN_FIT solution, except for the CREB column. Here, three subgraphs show a mismatch in scenarios 5, 10, and 15 (indicated by the left semicycles), while the other three show a mismatch in scenarios 6, 11, and 16 (indicated by the right semicycles).

The computation of all optimal subgraphs of the compressed network resulted in six solutions having the same minimal fitting error of 26 which has thus reduced much in comparison to 45 in the original model. Figure 5 shows a combined view of the six optimal solutions; the single solutions are shown in Table S1. In more detail, a positive influence of TGF*α* on STAT3 is not reflected in the measurements (see Figure 3); consequently, the edge TGF*α*→STAT3 is removed in all optimal solutions. Another edge that is removed in all solutions is PI3K→Rac_Cdc42, as a number of signals downstream of Rac_Cdc42 did not show the expected downregulated response to the PI3K inhibitor in the measurements (this is consistent with the results of the MCoS disussed in the previous subsection). Finally, by removing the edge ERK1/2→p70S6_1 in all solutions, the missing influence of MEK inhibitor on p70S6_1 is accommodated. The edges TGF*α*→MEK1/2 and Rac_Cdc42→MEK1/2 are only removed in some of the solutions. This is an example for two parallel routes that cannot be distinguished: the model structures containing both routes or either route give rise to the same sign-consistent labeling. In contrast, removing either of the edges p90RSK→CREB and p38→CREB results in different sign-consistent labelings, both showing the same number of discrepancies to the measurements: the phosphorylation state of CREB is neither affected by MEK inhibitor nor by p38 inhibitor. However, removing both edges at the same time would interrupt all routes from TGF*α* to CREB what is contradictory to the observed positive effect of TGF*α* in scenarios 1–6. Thus, in this case, allowing only the removal of edges is not sufficient to fully explain the observed measurements. This can be seen in Figure 6, where the two possible optimal sign-consistent labelings that SCEN_FIT would find for the six pruned model structures are shown in comparison to the discretized measurements: in each solution, there are three different remaining errors in the CREB column. The errors for STAT3 as well as the errors in response to PI3K inhibitor (scenarios 9 and 14) could be significantly reduced by removing the respective edges.

### Applying OPT_GRAPH

Next, we use the OPT_GRAPH procedure to identify edges that may be missing from the EGFR network and whose addition would therefore improve the goodness of fit to the data. Table 3 displays the edges that lead to the highest improvement as determined by OPT_GRAPH. All these edges have in common that they give rise to an additional route from TGF*α* to CREB not running over p38 or MEK1/2. By adding any of these edges to the model structure before reapplying the OPT_SUBGRAPH procedure, we can further reduce the fitting error to 23 (compared to 26 if only edge removals are allowed).

**Table 3:** Suggestions for new edges as computed by OPT_GRAPH.

tgfa→creb
jnk→creb
p70s61→creb
rac_cdc42→creb
tgfa→erk12
jnk→erk12
rac_cdc42→erk12

Adding any of these edges to the model structure leads to a decrease of the fitting error from 26 to 23.

**Figure 7:**
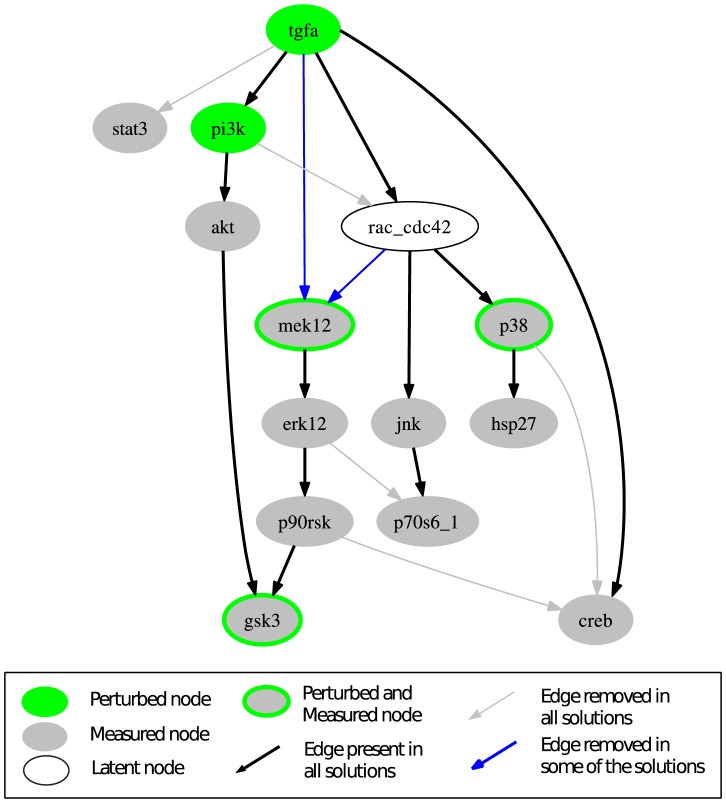
Combined view of the three optimal subgraphs resulting when adding TGF*α* to CREB to the initial model structure. In all three solutions, the edges erk12→p70s6_1, tgfa→stat3, p90rsk→creb and p38→creb are removed. Edges tgfa→mek12 and rac_cdc42→mek12 represent alternative pathways; at least one of both must be contained.

As an example, we show the optimized model structures when adding the edge TGF*α*→CREB. A combined view of the three optimal solutions (that can be found by OPT_GRAPH after adding this edge) is shown in Figure 7. As it was the case for the optimization in the original network, the edges TGF*α*→STAT3, PI3K→Rac_CDC42 and ERK1/2→p70S6_1 are removed in all solutions, while the edges TGF*α*→MEK1/2 and Rac_Cdc42→MEK1/2 are two alternative routes (either both are present or at least one of both; this gives the three optimal subgraphs). With the added edge TGF*α*→CREB the model structure comprises an activation route from TGF*α* to CREB that is independent of p38 and p90RSK, and removing both the p90RSK→CREB and p38→CREB edge in all solutions is now optimal.

**Figure 8:**
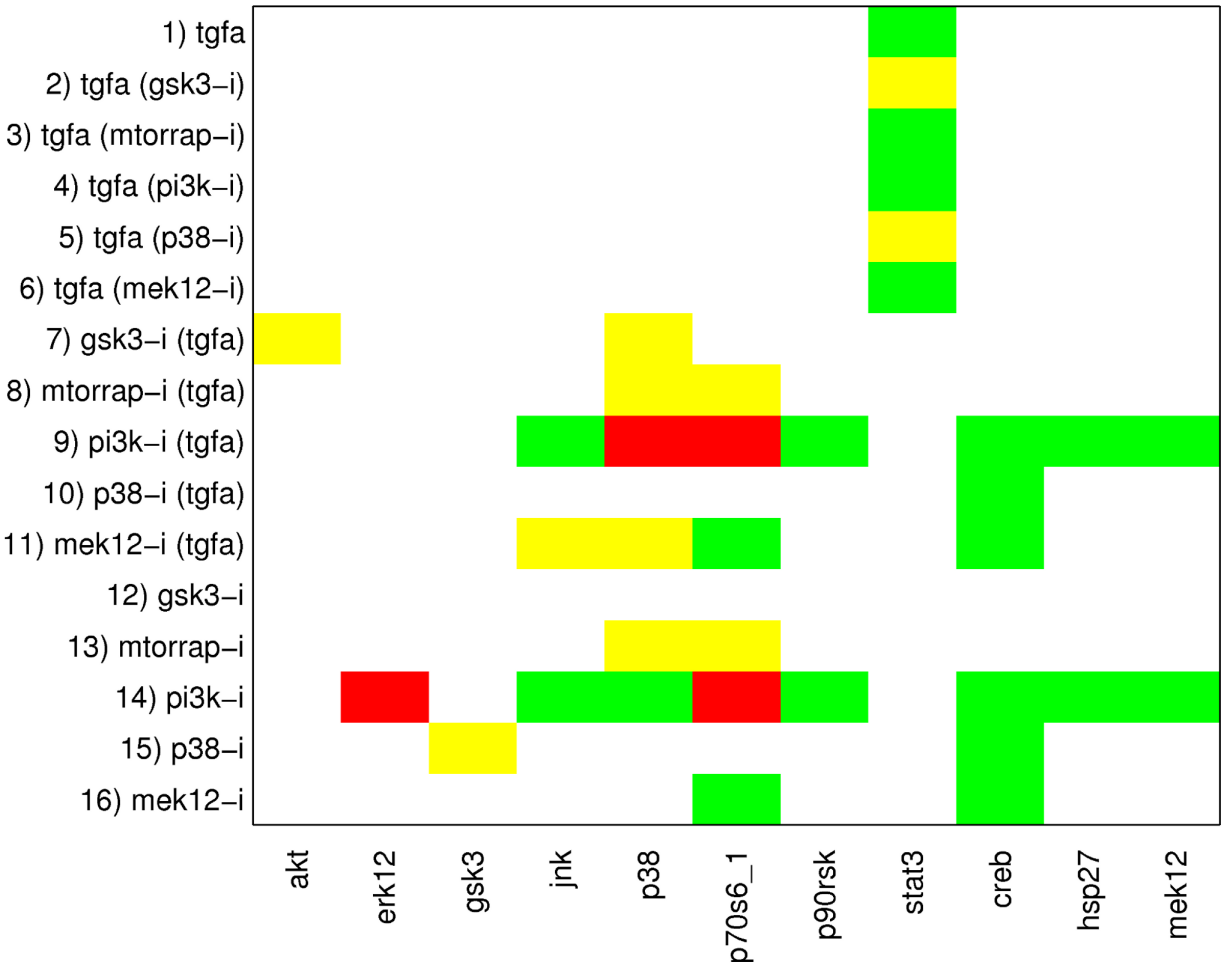
Comparison of the fitting errors of the initial model structure (see Figures 3 and 4) and of the optimal interaction graph shown in Figure 7. Green fields indicate an error that has been present in the original model structure, but could be removed by optimizing the model structure. Yellow fields refer to errors that could not be resolved, and red fields indicate errors that have not been present in the original model structure, but were introduced by the optimization.

All three solutions induce the same optimal sign-consistent node labeling. Figure 8 shows the mismatches of the experimental data in the optimal graph (Figure 7) vs. the mismatches in the initial model structure (Figure 4B). The measurements for CREB are now in full accordance with the model structure and the errors for STAT3 could be significantly reduced. Furthermore, a number of errors in scenarios 9 and 14 showing the influence of PI3K inhibitor could be eliminated, although at the same time a few mismatches for some nodes have been introduced. Finally, the influence of MEK inhibitor on p70S6_1 is now predicted correctly. Here, we considered only the addition of a single edge to improve the fit to data. In principle, one could remove all remaining discrepancies by adding further edges. However, in particular if the measurements show inconsistencies (e.g., the different effect of PI3K inhibitor on ERK1/2 with/without TGF*α*), some errors can only be removed by introducing a positive and a negative edge between a pair of nodes. Furthermore, edges leading only to a minor improvement of the fitting error are unlikely to represents a real effect. We also emphasize that proposed new edges may often indicate *indirect* rather than direct effects (representing then (hidden) paths in the network). In any case, dedicated experiments are required to confirm or prove the suggested causal links.

To summarize, essential findings of the network structure optimization in the EGFR/ErbB network—which may indicate important specifics of this signaling pathway in hepatocytes—are: (1) STAT3 is not activated by TGF*α*; (2) Phosphorylation of the autocatalytic domain of p70S6 (termed p70S6_1 in the model) is independent of ERK1/2; (3) The activation of CREB in response to TGF*α* is likely to be caused by a p38 and MEK1/2 independent route; and (4) The activation of Rac/Cdc42 is independent of PI3K activity. These results, generated in an automated way, confirm several of the conjectures formulated in [18] that were derived by inspection only. In addition, by identifying parallel activation routes that cannot be distinguished with the experimental data at hand, the presented approach contributes to a better understanding of the network topology and helps to suggest further experiments for uncovering the true wiring diagram of this important signaling pathway in the given cell type.

### Evaluation of the runtime behavior with respect to different problem sizes

When applying the four fundamental optimization problems SCEN_FIT, MCoS, OPT_SUBGRAPH and OPT_GRAPH to the EGFR/ErbB case study, we observed that all problems for both finding single and enumerating all solutions could be solved in a few seconds (see Figure S3), although hundreds or (in case of OPT_SUBGRAPH and OPT_GRAPH) even thousands of integer variables and constraints might be involved. However, since ILPs are in general NP-hard problems, we tested the runtime behavior more systematically by means of benchmarks to provide information on scalability and the ability of the algorithms to tackle larger, more complex problems. The benchmarks shown in Figure S3 evaluate the runtime of the formulations for problems of different size. Four experimental/simulated datasets were used: (i) the EGFR dataset interrogated throughout this paper, (ii) a random dataset of equal size to the EGFR dataset, (iii) a random dataset with the same number of signals (readouts) as the EGFR dataset but with double the number of experimental scenarios, and (iv) a random dataset with equal number of scenarios as the EGFR dataset, but with more measured signals. Moreover, four networks of different size were interrogated: (i) the compressed EGFR network (numbering 18 edges; Figure 4B), (ii) the uncompressed EGFR network, after removing non-observable and non-controllable parts of it (numbering 67 edges; Figure 4A), and two partially compressed networks, (iii) one numbering 32 edges, and (iv) one numbering 42 edges. For a detailed report on the benchmarks see Text S3; here, we give a brief overview of the results.

First of all, the benchmarks clearly showed a significant effect of the compression of the interaction graph as the amount of required memory and the runtimes were greatly decreased. All four problems (also in enumeration mode) could be solved within seconds for all training datasets. This is not only due to smaller network size (and thus fewer constraints and variables), but also due to the fact that the number of alternate optimal solutions to be found in the enumeration procedure, in particular for the OPT_SUBGRAPH problem, is strongly reduced.

Figure S3 shows that *single solutions* could be found within seconds for almost all problems, also in the larger networks. However, the runtime rapidly increased for OPT_SUBGRAPH and OPT_GRAPH problems when interrogated with the random dataset with double the number of experimental scenarios. The utilization of randomly generated data mimics a noisy dataset full of internal conflicts (i.e., the signal does not follow certain motifs like the actual data, but signals that are co-regulated in one scenario are anti-regulated in the next). This slows down the formulation and the runtime increases drastically, especially for the uncompressed network.

Regarding the *full enumeration* of alternate optimal solutions, we observed that all optimal SCEN_FIT and MCoS solutions could be found for all problem sizes within seconds. As expected, full enumeration of the optimal OPT_SUBGRAPH solutions (as well as solving the OPT_GRAPH problem) becomes challenging in larger networks for two reasons: (i) more than 17,000 variables and 37,000 constraints might be required to represent the problem, and (ii) a large number of alternate optimal solutions might exist. For this reason, several runs stopped because either the limit of the maximal number of solutions or the time limit was exceeded.

## Discussion

We presented a new framework for interrogating and training signaling networks based on measurements from stimulus-response experiments. Our approach represents signaling networks as interaction graphs and can thus immediately be applied to network topologies stored in many databases without the need to convert these graphs into other modeling formalisms. Interaction graphs capture merely the positive and negative edges between the components in the network; however, this information already sets constraints on the possible qualitative behavior of the nodes when stimulating or perturbing the network. Our approach uses Integer Linear Programming to encode these constraints and to predict the possible changes (down, neutral, up) of the activation levels of the involved players for a given experiment. Based on this ILP formulation we presented four basic optimization routines useful to detect and remove inconsistencies between measurements and predicted behaviors:

1. SCEN_FIT: Determination of a causal explanation for the measured activation changes of readout nodes under a given perturbation scenario. If the measurements are inconsistent with the network topology, the closest feasible explanation is identified.
2. Minimal Correction Sets: In case of an inconsistent scenario, determination of a minimal set of nodes whose states need to be corrected to make a single inconsistent scenario consistent.
3. OPT_SUBGRAPH: Determination of an optimal subgraph of a given network topology that can reflect the measurements for a set of scenarios at best.
4. OPT_GRAPH: Identification of edge candidate(s) whose insertion would improve the consistency of the graph with respect to a set of experimental scenarios the most.

The first two optimization problems seek to match the network topology with measurements from a *single* stimulus-response experiment. In contrast, (3) and (4) operate on a *set* of scenarios and seek to optimize (train) the network structure over all scenarios by removing or/and adding edges. For the first three problems we also provided enumeration algorithms to find multiple or all solutions that solve the optimization problem equally well (e.g., for problem (3), all optimal subgraphs that minimize the number of inconsistencies between measurements and predictions). The enumeration of all solutions is necessary to allow one to draw general conclusions, for example, that a certain edge is removed in all (not only in some) optimal solutions. However, the enumeration of optimal solutions may quickly become prohibitive in larger networks. We therefore employ effective compression techniques to deal with the combinatorial complexity arising in large-scale networks. In fact, this allowed us to also address the enumeration of multiple optimal solutions in the EGFR/ErbB case study where all performed computations could be finished within seconds on a standard PC. To assess the runtime behavior and scalability of our algorithms, we performed further benchmark tests showing that finding single optimal solutions to the four basic problems is feasible also in larger networks, whereas enumeration of all solutions, in particular for OPT_SUBGRAPH, becomes challenging (see Figure S3 and Text S3).

In contrast to the globally optimal solutions that will be delivered for problems (1)–(3), the identification of (a set of) missing edges reducing the fitting error the most (problem (4)) is based on a greedy algorithm which may deliver local instead of globally optimal solutions when adding more than one edge. However, given the huge search space of potentially missing (sets of) edges, the employed greedy algorithm appears to be a suitable and useful heuristics to suggest missing interactions in the IG model. If only one candidate edge is to be added (instead of a set), it even delivers the globally optimal solution, also in large networks.

To the best of our knowledge, our presented approach is the first that uses Integer Linear Programming directly on *interaction graphs* to systematically interrogate and train the wiring diagrams of signaling networks. Our framework shares some similarities with the approach of Saez-Rodriguez et al. [7] for which recently also an ILP formulation was conceived [29]. This method also starts with an IG representing the prior knowledge; however, the IG is then translated to a superstructure of Boolean networks within which the optimal (sub)model fitting the data at best is identified. Although a correctly reconstructed Boolean network can potentially provide a more specific view on the network structure than an IG, the search space is considerably larger since usually a vast number of possible Boolean networks can be constructed from a given IG. This may lead to highly underdetermined problems and enumeration strategies as discussed herein can become intractable. Furthermore, Boolean networks require a strict binarization of the nodes' states whereas in the IG formulation we consider “influences”. This may lead to different results. For example, the Boolean function for a node 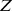 may read 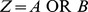. Assume that we consider the influence of (external) activation of node 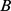 given the network state where 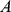 is active and 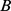 inactive; hence, where 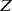 is already in the active state. The Boolean model will tell us that 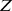 remains in state 1 when activating 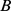, hence, the influence of 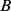 seems to be not relevant. However, Boolean functions are discrete approximations of the true mechanisms and what one could probably see in the measurements is that the level of 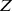 goes from “high” to “very high”. In the IG, we can still account for this effect stating that an elevated level of 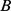 induces a positive effect on 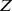. So discretized node states need not to be considered in the IG model; however, similar as for the Boolean model, some kind of discretization of the data will be required as well when classifying a change of an activation level to be significant or not. Finally, we also mention that methods for the enumeration of solutions and for the search for missing edges were not presented in [29].

The approach that is arguably closest to ours is the method introduced in [11]–[13]. This framework is also based on IG and uses a similar consistency rule as we did herein. However, there are a number of key differences. First, we explicitly allow a “0” change to mark non-affected states of nodes. This extension seems to be essential, for example, when perturbation of a node 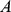 cannot affect another node 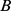 simply because (in the true topology) a path from 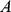 to 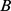 does not exist. Second, the four basic problem formulations presented herein go beyond the techniques introduced in [11]–[13]. In particular, the training of the topology, that is, the identification of inactive or missing interactions based on a library of stimulus-response experiments, was not considered in these works. A third key difference is that we formulated the constraints resulting from the consistency rules as an ILP problem, whereas [13] uses Answer Set Programming (ASP). Both ILP and ASP deliver globally optimal solutions and highly optimized solvers exist. Using ILP or ASP solvers is not straightforward for non-experts and with *SigNetTrainer* we provide an easy-to-use toolbox. However, it would be an interesting aspect for future work to compare ASP and ILP formulations of the training and enumeration problems formulated herein.

We demonstrated the power of our proposed approach by interrogating and (re-)training a manually curated IG model of EGFR/ErbB signaling against a library of high-throughput phosphoproteomic data measured in primary human hepatocytes. Our algorithms could systematically uncover all inconsistencies between measurements and network topology and gave possible explanations for them. Novel biological insights for this important signaling pathway could be revealed by listing interactions that are likely to be inactive in hepatocytes and by giving suggestions for possibly missing interactions that, if included, would significantly improve the goodness of fit. Clearly, these predictions await experimental validation.

This study gave a proof of principle for our methodology, showing its flexibility and that it can be applied to a wide range of problems arising when confronting signaling network topologies with experimental datasets. Given that only fairly accessible biological knowledge is required and that all related algorithms were implemented in a freely available toolbox make it an appealing approach for various applications.

## Supporting Information

Figure S1 **Raw training data.** A subset of the phosphoprotein data published in [2], capturing the signaling response of primary human hepatocytes to TGF*α* in combination with six specific molecular inhibitors (including the no-inhibitor treatment): MEK12-i, p38-i, PI3K-i, mTORrap-i, GSK3-i, no-inhib. Each subplot shows the phosphorylation state of the respective protein in fluorescent units (obtained via xMAP technology), measured 0 minutes (left border) and 25 minutes (right border) after stimulation.
(EPS)

Figure S2
**Cumulative fitness error of optimal SCEN_FIT solutions over all 16 scenarios in the (compressed) EGFR/ErbB network as a function of the two discretization thresholds.** The cumulative fitness error of optimal SCEN_FIT solutions over all 16 scenarios in the (compressed) EGFR/ErbB network as a function of the significant increase and significant decrease thresholds is plotted. The thresholds combination used for all analyses presented in this paper are plotted as a blue rectangle. There is a relatively broad range for “significant decrease” in 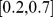 and “significant increase” in 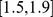 where the fitness error assumes its lowest values (40–50). Outside that area the fitness error increases rapidly. The thresholds used in the EGFR/ErbB study (0.66 and 1.5, respectively) are inside that range and result in a total fitness error of 45 (see Figure 3 in main text).(PNG)

Figure S3 **Evaluation of runtimes of** ***SigNetTrainer*** **(GUROBI version) with respect to the four basic optimization problems and different problem sizes.** Runs for all four ILP problems introduced in this paper (SCEN_FIT, MCoS, OPT_SUBGRAPH, OPT_GRAPH) are shown in the corresponding columns. For each run the CPU time, number of variables, number of constraints, and number of found solutions are reported, both for obtaining a single solution and for enumeration of solutions. The first five columns give a description of each run regarding the interrogated data and network: the dataset used (EGFR data, random data, more scenarios, more signals; see explanations in the main text and in text S3), the number of reactions in the network (18, 32, 42, 67), number of measured signals, number of scenarios and number of inputs. A time limit is set for each run at 64,000 seconds. For the enumeration benchmarks, a maximum number of allowed solutions is set at 1000 solutions. The maximum allowed memory is 4 GB. Instances where the algorithm did not complete the run due to time-out are marked with red. All calculations were done on a PC with a 2.2 GHz Intel quad core i7 CPU (only a single core was used) and 4 GB 1333 MHz DDR3 memory. The default optimality tolerance was used in all optimizations for the GUROBI solver (see also http://www.gurobi.com/documentation/5.0/reference-manual/).
(PDF)

Table S1 **Optimal model structures derived from the compressed EGFR/ErbB model by OPT_SUBGRAPH with enumeration.**
(PDF)

Text S1 **Getting started with** ***SigNetTrainer*** **.**
(PDF)

Text S2 **Sensitivity analysis of the SCEN_FIT solutions with respect to the chosen discretization thresholds.**
(PDF)

Text S3 **Systematic evaluation of ILP runtimes with respect to problem size.**
(PDF)
