# Supplementary figures and images for: Detecting and Removing Inconsistencies between Experimental Data and Signaling Network Topologies Using Integer Linear Programming on Interaction Graphs
Source: PLoS Comput Biol. 2013 Sep 5;9(9):e1003204. doi: 10.1371/journal.pcbi.1003204 (PMC3764019; doi:10.1371/journal.pcbi.1003204)

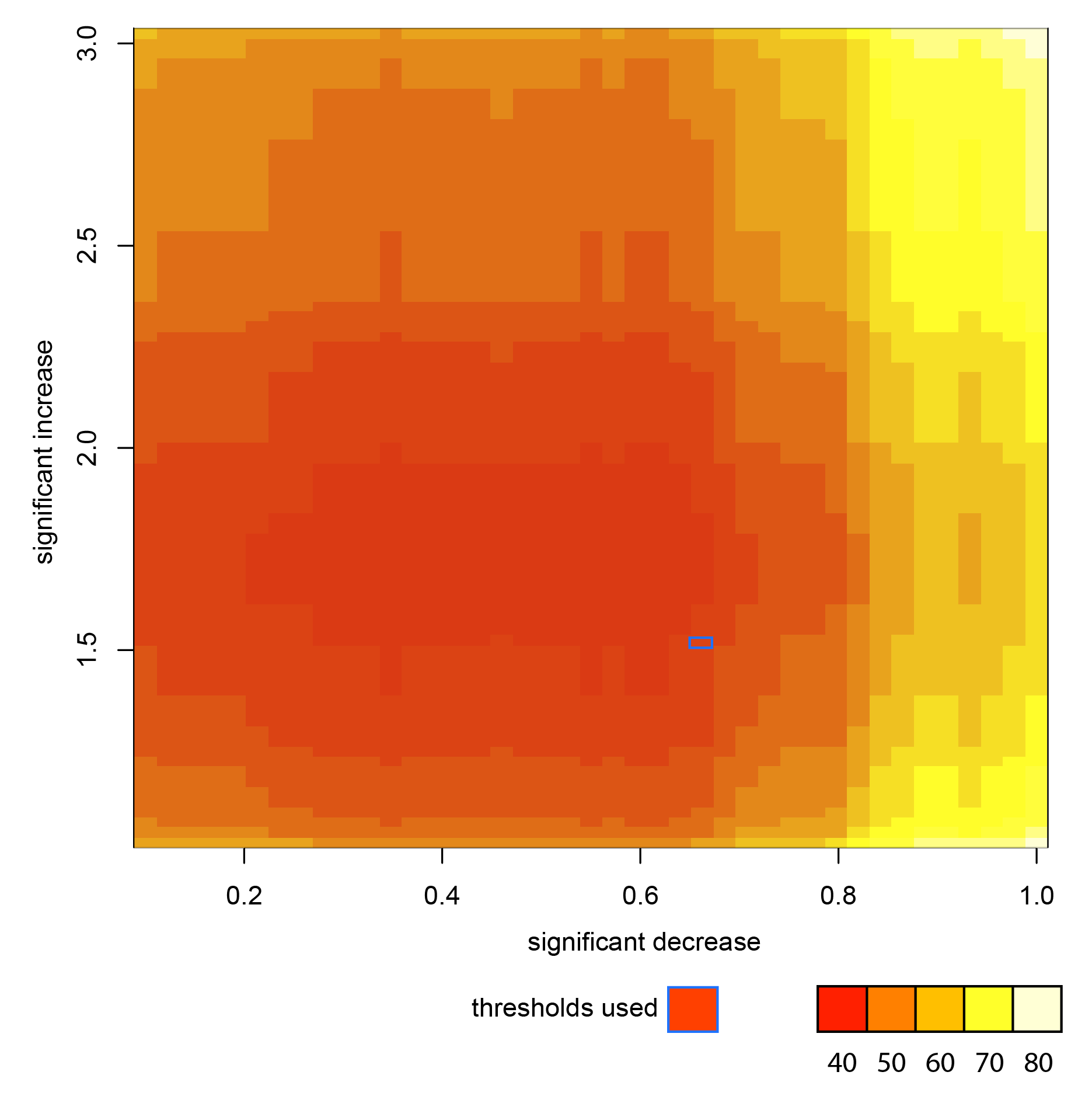

Supplement: Figure S2 — Cumulative fitness error of optimal SCEN_FIT solutions over all 16 scenarios in the (compressed) EGFR/ErbB network as a function of the two discretization thresholds. The cumulative fitness error of optimal SCEN_FIT solutions over all 16 scenarios in the (compressed) EGFR/ErbB network as a function of the significant increase and significant decrease thresholds is plotted. The thresholds combination used for all analyses presented in this paper are plotted as a blue rectangle. There is a relatively broad range for “significant decrease” in and “significant increase” in where the fitness error assumes its lowest values (40–50). Outside that area the fitness error increases rapidly. The thresholds used in the EGFR/ErbB study (0.66 and 1.5, respectively) are inside that range and result in a total fitness error of 45 (see Figure 3 in main text). (PNG) [file pcbi.1003204.s002.png]
