# Supplementary material for: Detecting and Removing Inconsistencies between Experimental Data and Signaling Network Topologies Using Integer Linear Programming on Interaction Graphs
Source: PLoS Comput Biol. 2013 Sep 5;9(9):e1003204. doi: 10.1371/journal.pcbi.1003204 (PMC3764019; doi:10.1371/journal.pcbi.1003204)
Supplement: Figure S3 — Evaluation of runtimes of SigNetTrainer (GUROBI version) with respect to the four basic optimization problems and different problem sizes. Runs for all four ILP problems introduced in this paper (SCEN_FIT, MCoS, OPT_SUBGRAPH, OPT_GRAPH) are shown in the corresponding columns. For each run the CPU time, number of variables, number of constraints, and number of found solutions are reported, both for obtaining a single solution and for enumeration of solutions. The first five columns give a description of each run regarding the interrogated data and network: the dataset used (EGFR data, random data, more scenarios, more signals; see explanations in the main text and in text S3), the number of reactions in the network (18, 32, 42, 67), number of measured signals, number of scenarios and number of inputs. A time limit is set for each run at 64,000 seconds. For the enumeration benchmarks, a maximum number of allowed solutions is set at 1000 solutions. The maximum allowed memory is 4 GB. Instances where the algorithm did not complete the run due to time-out are marked with red. All calculations were done on a PC with a 2.2 GHz Intel quad core i7 CPU (only a single core was used) and 4 GB 1333 MHz DDR3 memory. The default optimality tolerance was used in all optimizations for the GUROBI solver (see also http://www.gurobi.com/documentation/5.0/reference-manual/). (PDF) [file pcbi.1003204.s003.pdf]

		#reactions	#signals	#scenarios	#inputs	SCEN _FIT				MCoS			
						CPU time	#variables	#constraints	#solutions	CPU time	#variables	#constraints	#solutions
Single Solution	EGFR data	18	11	1	2	< 0.01	179	368.75	1	< 0.01	207	369.75	1
		32	11	1	2	< 0.01	292	608.75	1	0.01	330	609.75	1
		42	11	1	2	< 0.01	377	788.75	1	0.01	425	789.75	1
		67	11	1	2	0.01	597	1253.75	1	0.04	657	1254.75	1
	random data	18	11	1	2	< 0.01	179	368.75	1	< 0.01	207	369.75	1
		32	11	1	2	< 0.01	292	608.75	1	0.01	330	609.75	1
		42	11	1	2	< 0.01	377	788.75	1	0.01	425	789.75	1
		67	11	1	2	0.01	597	1253.75	1	0.04	657	1254.75	1
	more signals	18	13	1	2	< 0.01	181	372.75	1	< 0.01	209	373.75	1
		32	18	1	2	< 0.01	299	622.75	1	< 0.01	337	623.75	1
		42	23	1	2	< 0.01	389	812.75	1	< 0.01	437	813.75	1
		67	38	1	2	< 0.01	624	1307.75	1	< 0.01	702	1308.75	1
Enumeration	EGFR data	18	11	1	2	< 0.01	179	368.75	1	< 0.01	207	369.87	1.06
		32	11	1	2	< 0.01	292	608.75	1	< 0.01	330	611.06	1.87
		42	11	1	2	< 0.01	377	788.75	1	0.02	425	791.12	1.93
		67	11	1	2	< 0.01	597	1253.75	1	0.16	675	1258.44	4.06
	random data	18	11	1	2	< 0.01	179	368.75	1	< 0.01	207	371.812	2.56
		32	11	1	2	< 0.01	292	608.75	1	0.05	330	616.56	7.06
		42	11	1	2	< 0.01	377	788.75	1	0.29	425	806.81	17.12
		67	11	1	2	0.01	597	1253.75	1	3.36	675	1298.06	43.31
	more signals	18	13	1	2	< 0.01	181	372.75	1	< 0.01	209	373.75	1
		32	18	1	2	< 0.01	299	622.75	1	< 0.01	337	623.75	1
		42	23	1	2	< 0.01	389	812.75	1	< 0.01	437	813.75	1
		67	38	1	2	< 0.01	624	1307.75	1	< 0.01	702	1308.75	1

		#reactions	#signals	#scenarios	#inputs	OPT _SUBGRAPH				OPT _GRAPH			
						CPU time	#variables	#constraints	#solutions	CPU time	#variables	#constraints	#tested edges
Single Solution	EGFR data	18	11	16	6	0.08	2594	5612.00	1	19.40	2691	5837.40	298
		32	11	16	6	0.09	4192	9228.00	1	72.46	4289	9453.07	536
		42	11	16	6	0.10	5402	11948.00	1	283.89	5499	12172.90	860
		67	11	16	6	0.22	8547	18988.00	1	30879.20	8644	19212.50	2351
	random data	18	11	16	6	0.06	2594	5612.00	1	75.31	2691	5837.40	298
		32	11	16	6	0.22	4192	9228.00	1	5076.05	4289	9453.07	536
		42	11	16	6	0.45	5402	11948.00	1	63662.5	5499	12172.90	860
		67	11	16	6	10.62	8547	18988.00	1	>64000	8644	19212.00	XXXX
	more scenarios	18	11	32	6	0.22	5170	11200.00	1	1740.87	5363	11650.80	298
		32	11	32	6	4.76	8352	18432.00	1	>64000	8545	18881.90	XXXX
		42	11	32	6	26.95	10762	23872.00	1	>64000	10955	24320.00	XXXX
		67	11	32	6	1404.15	17027	37952.00	1	>64000	17220	38400.00	XXXX
	more signals	18	13	16	6	0.07	2626	5676.00	1	44.51	2723	5901.40	298
		32	18	16	6	0.31	4304	9452.00	1	8134.02	4401	9677.07	536
		42	23	16	6	0.47	5594	12332.00	1	>64000	5691	12556.80	XXXX
		67	38	16	6	1.78	8979	19852.00	1	>64000	9076	20076.00	XXXX
Enumeration	EGFR data	18	11	16	6	0.38	2594	5618.00	6	NA	NA	NA	NA
		32	11	16	6	351.97	4192	9728.50	>1000	NA	NA	NA	NA
		42	11	16	6	506.61	5402	12448.50	>1000	NA	NA	NA	NA
		67	11	16	6	1083.83	8547	19488.50	>1000	NA	NA	NA	NA
	random data	18	11	16	6	30.42	2594	5828.00	216	NA	NA	NA	NA
		32	11	16	6	527.20	4192	9728.50	>1000	NA	NA	NA	NA
		42	11	16	6	773.4	5402	12448.50	>1000	NA	NA	NA	NA
		67	11	16	6	3836.3	8547	19488.50	>1000	NA	NA	NA	NA
	more scenarios	18	11	32	6	8.98	5170	11204.9	8	NA	NA	NA	NA
		32	11	32	6	26687.3	8352	18931.00	>1000	NA	NA	NA	NA
		42	11	32	6	>64000	10762	23896.00	XXXX	NA	NA	NA	NA
		67	11	32	6	>64000	17027	37953.00	XXXX	NA	NA	NA	NA
	more signals	18	13	16	6	245.95	2626	6176.50	>1000	NA	NA	NA	NA
		32	18	16	6	415.46	4304	9952.50	>1000	NA	NA	NA	NA
		42	23	16	6	1256.00	5594	12832.5	>1000	NA	NA	NA	NA
		67	38	16	6	9886.82	8979	20352.50	>1000	NA	NA	NA	NA
