# Supplementary material for: Detecting and Removing Inconsistencies between Experimental Data and Signaling Network Topologies Using Integer Linear Programming on Interaction Graphs
Source: PLoS Comput Biol. 2013 Sep 5;9(9):e1003204. doi: 10.1371/journal.pcbi.1003204 (PMC3764019; doi:10.1371/journal.pcbi.1003204)
Supplement: Table S1 — Optimal model structures derived from the compressed EGFR/ErbB model by OPT_SUBGRAPH with enumeration. (PDF) [file pcbi.1003204.s004.pdf]

Table S1

**Removed edges**	Solution 1	Solution 2	Solution 3	Solution 4	Solution 5	Solution 6
pi3k→rac_cdc42	1	1	1	1	1	1
rac_cdc42→mek12	1		1			
rac_cdc42→p38						
tgfa→rac_cdc42						
tgfa → pi3k						
tgfa → mek12				1	1	
pi3k → akt						
p90rsk → creb	1	1		1		
p90rsk → gsk3						
akt → gsk3						
rac_cdc42 → jnk						
p38 → hsp27						
p38 → creb			1		1	1
mek12 → erk12						
jnk → p70s6_1						
erk12 → p70s6_1	1	1	1	1	1	1
erk12 → p90rsk						
tgfa → stat3	1	1	1	1	1	1

Table S1. Optimal model structures derived from the compressed EGFR/ErbB model by OPT_SUBGRAPH with enumeration. The complete set of optimal subgraphs that fit the measured data in Figure 3 is shown. The first column shows the complete list of edges included in the compressed network. Columns 2–7 denote with an entry of "1" whether the respective edge was removed by the optimization procedure.
