# Supplementary material for: Detecting and Removing Inconsistencies between Experimental Data and Signaling Network Topologies Using Integer Linear Programming on Interaction Graphs
Source: PLoS Comput Biol. 2013 Sep 5;9(9):e1003204. doi: 10.1371/journal.pcbi.1003204 (PMC3764019; doi:10.1371/journal.pcbi.1003204)
Supplement: Text S1 — Getting started with SigNetTrainer . (PDF) [file pcbi.1003204.s005.pdf]

Getting started with *SigNetTrainer*

Part 1: *SigNetTrainer* for MATLAB/CPLEX and MATLAB/GLPK

Part 2: *SigNetTrainer* for GUROBI

Download: `http://www.mpi-magdeburg.mpg.de/projects/cna/etcdownloads.html`

Part 1: *SigNetTrainer* for MATLAB/CPLEX and MATLAB/GLPK

1 Introduction

This first part of the present document is the manual of *SigNetTrainer* for MATLAB/CPLEX and MATLAB/GLPK. *SigNetTrainer* is a toolbox dedicated for interrogating and training signaling networks (represented as interaction graphs) with experimental data from stimulus-response experiments. *SigNetTrainer* uses Integer Linear Programming (ILP) and is based on the methods described in the main text of this publication. The MATLAB-based version of the toolbox (presented herein) was developed by S. Klamt; it runs under MATLAB and uses either the IBM ILOG CPLEX Optimizer or GLPK/GLPKMEX for solving the ILP problems. An alternative implementation of *SigNetTrainer* based on C-files and GURBOI was developed by I.N. Melas (see second part of this document). All versions of *SigNetTrainer* are freely available at the web-site: `http://www.mpi-magdeburg.mpg.de/projects/cna/etcdownloads.html`.

The basic functionality of *SigNetTrainer* can be briefly described as follows. Given an interaction graph topology (stored in a file *netfile*) and a set of experiments in each of which some nodes were perturbed (defined in a file *pertfile*) and the resulting qualitative response (up, down, unchanged) of some nodes were measured (defined in a file *measfile*), four basic problems can be addressed by *SigNetTrainer*:

(1) SCEN_FIT: Determine a causal explanation for the measured activation changes of readout nodes for a given perturbation experiment. If the measurements are inconsistent with the network topology, find the closest consistent sign pattern for the nodes.

(2) Minimal Correction Sets (MCoS): In case of an inconsistent scenario, what is a minimal set of nodes that need to be corrected (by artificial external perturbations) to make the inconsistent scenario consistent.

(3) OPT_SUBGRAPH: Determine an optimal subgraph of the given network topology that can fit the measurements for a set of scenarios the best.

(4) OPT_GRAPH: Identify edge candidate(s) whose insertion (in combination with removal of existing edges) would improve the consistency of the graph with respect to a set of experimental scenarios the most.

The first two optimization problems seek to match the network topology with measurements from a single stimulus-response experiment. In contrast, (3) and (4) operate on a *set* of scenarios and seek to optimize (train) the network structure over all scenarios, either by removing or/and by adding edges. For the first three problems *SigNetTrainer* also provides enumeration algorithms to find multiple or all solutions that solve the opti-

mization problem equally well (e.g., for problem (3), all optimal subgraphs that minimize the number of inconsistencies between measurements and predictions).

SigNetTrainer for MATLAB/CPLEX and MATLAB/GLPK provides to each of the four problems above a MATLAB function which can be used for both finding a single optimal solution or enumerating all of them. When calling these functions the user can define the solver to be used (either CPLEX via its MATLAB connector or GLPK via GLPKMEX) which needs to be installed beforehand. We tested our implementation with CPLEX 12.4 and the latest GLPKMEX 2.11 (both on a 64-bit LINUX system).

GLPKMEX is freely available at: `http://sourceforge.net/projects/glpkmex/`
Academic users can also obtain a free academic version of CPLEX, see:
`http://www-03.ibm.com/ibm/university/academic/pub/page/membership`.

Generally, both solvers work well (ensure that you use the latest GLPKMEX version!). However, for large-scale problems we observed that CPLEX may perform much faster and more stable than GLPKMEX (in one example, CPLEX finished after seconds whereas GLPKMEX did not finish after 24 hours). So if you have the choice then we strongly recommend CPLEX.

SigNetTrainer also includes a MATLAB routine for network compression and a function for loading the files related to a project. In addition, example files for the EGF study discussed in the main text are provided with the package.

Note that the present manual will describe usage of the available algorithms rather than re-describing the theory behind the methods.

If you encounter any technical problems please contact S. Klamt:
klamt@mpi-magdeburg.mpg.de.

2 Files

SigNetTrainer for MATLAB/CPLEX and MATLAB/GLPK comes with the following files:

MATLAB functions:

- **scenfit.m**: for solving SCEN_FIT problems.
- **mcos.m**: for computing Minimal Correction Sets.
- **optsubgraph.m**: for solving OPT_SUBGRAPH problems.
- **optgraph.m**: for solving OPT_GRAPH problems.
- **compressIG.m**: for compressing the network (as an optional preprocessing step).

- **loadSigProject.m**: function for reading the network file (*netfile*) and for importing the signaling data (*pertfile* and *measfile*) resulting in a *SigProject* variable.
- **CNASFNetwork2sif.m**: function required by loadSigProject.m
- **CNAsif2SFNetwork.m**: function for saving a compressed network (as obtained by compressIG) as *netfile* in SIF format that can later be read by *loadSigProject.*

manual.pdf: This manual.

EGFexample: A directory containing the files for the EGFR/ErbB example studied in the Results section of the main text. You may recompute the results presented therein by using these files as input for *SigNetTrainer* (usage is explained in section 4).

MinExample: A directory containing *netfile, pertfile* and *measfile* of the simple network example discussed in the Introduction section of the main text (just for playing and testing).

3 Installation

3.1 System Requirements and Initialization

To use *SigNetTrainer* for MATLAB/CPLEX or MATLAB/GLPK you need to install

(1) MATLAB;

(2) an ILP solver:

  (2a) either IBM ILOG CPLEX Optimizer (to be called via its MATLAB connector; the (only) used CPLEX function is *cplexmilp*

  (2b) or GLPK to be called via GLPKMEX (*SigNetTrainer* calls glpk.m which itself calls the MEX file glpkcc);

(3) the MATLAB toolbox *CellNetAnalyzer* which is needed by compressIG.m, loadSigProject.m, optgraph.m, CNASFNetwork2sif.m, and CNAsif2SFNetwork.m.

CPLEX Optimizer is freely available for acedemic research institutes via:
`http://www-03.ibm.com/ibm/university/academic/pub/page/membership`.
and GLPKMEX is available under the GNU license via
`http://sourceforge.net/projects/glpkmex/`

CellNetAnalyzer can be downloaded for academic use from:
`http://www.mpi-magdeburg.mpg.de/projects/cna/cna.html`.

Once you have downloaded/installed all packages on your PC, start MATLAB. You first need to set the paths to the CPLEX library or/and to GLPKMEX (possibly also in the startup.m script). On my LINUX system I set the path to CPLEX by entering:

addpath('/usr/local/cplex/CPLEX_Studio124/cplex/matlab/')

For initializing *CellNetAnalyzer* (CNA) you need to change to CNA's main directory. Then enter:

startcna(1)

Change then back to the main directory of *SigNetTrainer*. You can now start working with the toolbox.

4 Creating and loading a Signaling Project

The function *loadSigProject* builds a signaling project variable (denoted herein by *SigProject*) which can subsequently be used by *SigNetTrainer*'s optimization routines. The syntax is:

SigProject = loadSigProject(netfile,pertfile,measfile)

netfile, *pertfile* and *measfile* are strings (character arrays) defining the names of three files that together specify a signaling project. These files must be created by the user (regarding the format see below). Here is an example call loading the EGF network and associated data studied in the Results section of the main text and provided in the 'EGFexample' folder:

SigProject=loadSigProject('EGFexample/EGFNetworkCompressed.txt',...
'EGFexample/EGFinputs.txt','EGFexample/EGFmeasurements.txt')

The *SigProject* struct variable returned by the function resembles a *CellNetAnalyzer* project variable. In particular, it contains the field *SigProject.specID* and *SigProject.reacID* storing the names of the species (nodes) and edges, respectively.

In the following we describe the format of the three input files:

netfile: defines the interaction graph in SIF file format (the file may have extension .sif but also .txt etc). With the SIF format, the interaction graph is defined as follows:

- Tab-delimited file
- The edges (reactions) of the interaction graph are given row-wise.
- First column contains the start nodes of the respective edge.
- Second column describes the edge sign which is either positive (indicated by "1") or negative (indicated by "-1").

- Third column contains the end node of the respective edge.
- No special characters (except “underscore”) are allowed in the node names.
- The names of signaling molecules must correspond to names used as stimuli/inputs in *pertfile* and for measured signals in *measfile*.

An example of a *netfile* is shown in Figure 2. Importantly, it is recommended that the network does not contain positive feedback loops (positive cycles) in the network to avoid ’self-explaining’ measurements (a future version will tackle positive cycles more appropriately).

jnk	1	p70s61
jnk	1	cjun
pp2a	-1	cfos
jnk	1	cfos
ip3	1	ca
grb2	1	gab1
grb2	1	pak1
gab1	1	shp2
gab1	1	pi3k
shp2	-1	rasgap
gab1	1	rasgap
erk12	1	p70s61
pdk1	1	p90rsk
erk12	1	p90rsk

Figure 1: Example of a *netfile*.

pertfile: defines the perturbations (e.g., inhibitors or ligands) used in the respective experimental scenarios. The format is as follows:

- Tab-delimited file
- First row contains the names of the corresponding input (=perturbed) nodes in the columns. Not all network nodes have to be given here (since normally not all nodes will be perturbed).
- Each subsequent row corresponds to a specific experimental scenario, where the columns give the (imposed) values of the perturbed nodes: “1” corresponds to up-regulation of the respective node, “-1” corresponds to down-regulation of the respective node, “0” corresponds to set the respective node to unchanged, and “nan” implies the respective node was not fixed (and thus free) in that scenario.
- No special characters are allowed in the names of the input nodes.
- The names of the perturbed nodes must be consistent with the corresponding node names in the *netfile*.

The *pertfile* of the (compressed) EGF network studied in the Results section of the main text is shown in Figure 3.

tgfa	mek12	p38	pi3k	gsk3
0	-1	nan	nan	nan
0	nan	-1	nan	nan
0	nan	nan	-1	nan
0	nan	nan	nan	-1
0	-1	nan	nan	nan
0	nan	-1	nan	nan
0	nan	nan	-1	nan
0	nan	nan	nan	-1
1	0	nan	nan	nan
1	nan	0	nan	nan
1	nan	nan	0	nan
1	nan	nan	nan	0
1	nan	nan	nan	nan

Figure 2: Example of a *pertfile*.

measfile: specifies the measured node changes (up, down, neutral) in the experiments (upon the perturbations specified in the *pertfile*). Note that the i-th row in the *measfile* gives the measurements to the i-th perturbation defined in the *pertfile*.

- Tab-delimited file
- First row contains the names of the measured signals (in the columns). Not all network nodes must be given (as not all nodes might be measurable).
- Subsequent rows correspond to different experimental scenarios, columns specify the measured signals: "1" corresponds to up-regulation, "-1" corresponds to down-regulation, "0" corresponds to unchanged signal, and "nan" implies the respective signal was not measured in that scenario.
- The names of the measured signals must be consistent with the corresponding signaling species in the *netfile* and *pertfile*.

The measfile of the (compressed) EGF network studied in the Results section of the main text is shown in Figure 4.

akt	erk12	gsk3	jnk	p38	p70s61	p90rsk	stat3	creb	hsp27	mek12
0	-1	-1	0	0	0	-1	0	0	0	-1
0	0	-1	0	-1	0	0	0	0	-1	0
-1	-1	-1	0	0	-1	0	0	0	0	0
0	0	-1	0	0	0	0	1	0	0	0
0	-1	0	-1	-1	0	-1	0	0	0	-1
0	0	0	0	-1	0	1	0	0	-1	0
-1	1	0	0	-1	-1	0	0	0	0	0
-1	0	-1	0	-1	0	0	0	0	0	0
1	1	1	1	1	1	1	0	1	1	0
1	1	1	1	0	1	1	-1	1	1	1
0	1	1	1	1	1	1	0	1	1	1
1	1	0	1	1	1	1	-1	1	1	1
1	1	1	1	1	1	1	0	1	1	1

Figure 3: Example of a *measfile*.

5 Compressing a network

For compressing a network (using the compression rules described in the Methods section of the main text) do the following:

(1) Load the network from a *netfile* by running:

    cnap=CNAsif2SFNetwork(netfile)

    Here, *netfile* is the name of the file describing the initial (uncompressed) graph. The network in *netfile* must be specified in SIF format as described in the previous section.

(2) Use MATLAB cell variables to specifiy the names of the perturbed nodes and of the (measured) readout nodes, for example

    readouts={'erk12','stat3'}

    perturb={'tgfa','p38'}

(3) Call the actual network compression routine by

    cnapnew=compressIG(cnap,readouts,perturb)

(4) Write the new network file (which can then later be used, e.g., by *loadSigProject* when loading a signaling network project:)

    CNASFNetwork2sif(cnapnew,netfile)

    Here, *netfile* is the name of the new file in which the compressed network file is stored.

6 Running the optimizations

Once a signaling project has been created and loaded by *loadSigProject* as described in section 4, we may now apply the four basic optimization routines to check/train the network topology against the experimental data.

6.1 SCEN_FIT

The SCEN_FIT procedure can be applied to single experiments (scenarios) is called as follows:

[solutions,optval]=scenfit(SigProject,scenario,numsol,solver)

Input arguments:

- *SigProject*: a *SigProject* variable as obtained from *loadSigProject* (described in section 4).
- *scenario*: specifies the number of the experiment (scenario) to be considered by SCEN_FIT. For example, if scenario=4 then the 4-th experiment from the pertfile/measfile is taken.
- *numsol*: How many optimal solutions are to be computed (default: 1).
- *solver*: Which solver shall be used. *solver*=1 $\rightarrow$ CPLEX, *solver*=2 $\rightarrow$ GLPK. If no solver is specified, CPLEX will be used as default. (For installation of the solvers see section 3).

The *scenfit* function delivers *numsol* many optimal solutions to the SCEN_FIT problem. The solutions are stored in the columns of the returned array *solutions*. Hence, this variable has dimension n x *numsol* (n = number of species). Each of these solutions provides a sign-consistent node pattern that has minimal distance to the measurements (the i-th row of *solutions* corresponds to the i-th node in the network; the name of the i-th node is stored in *SigProject.specID(i,:))*. The optimal (minimal) mismatch to the measurements is stored in the output argument *optval*. Note that it may happen that less then *numsol* many optimal solutions exist, in this case the array *solutions* will obviously contain less columns.

6.2 Minimal Correction Sets

Minimal Correction Sets (MCoS) can be computed for single experiments (scenarios) and the asscociated routine is called as follows:

[solutions,optval]=mcos(SigProject,scenario,numsol,solver)

Input arguments:

- *SigProject*: a *SigProject* variable as obtained from *loadSigProject* (described in section 4).
- *scenario*: specifies the number of the experiment (scenario) to be considered for MCoS calculation. For example, if scenario=4 then the 4-th experiment from the pertfile/measfile is taken.
- *numsol*: How many optimal solutions are to be computed (default: 1).
- *solver*: Which solver shall be used. *solver*=1 → CPLEX, *solver=2* → GLPK. If no solver was specified, CPLEX will be used as default. (For installation of the solvers see section 3).

The *mcos* function delivers *numsol* many optimal MCoSs in the columns of array *solutions*. The latter variable has thus dimension n x *numsol* (n = number of species). Each of these solutions provides a minimal number of (artficial/unknown) perturbations in certain species that will lead to a sign-consistent node pattern for the given inputs and measurements of the considered scenario. The value of the external perturbation (MCoS) value for a node can be 1 (positive input), -1 (negative input) or 0 (no additional input). The i-th row of *solutions* corresponds to the i-th node in the network, the name of this ndoe is stored in *SigProject.specID(i,:)).* The optimal (minimal) number of external corrections required in the optimal MCoS is stored in *optval.* Note that it may happen that less then *numsol* many optimal MCoSs exist, in this case the array *solutions* will obviously contain less columns.
The found MCoSs are also displayed during the computation.

6.3 OPT_SUBGRAPH

An OPT_SUBGRAPH problem usually relates to a network and a *set* of experiments (scenarios) and the goal is to find edge removals that minimize the mismatch between network topology and measurements. *SigNetTrainer*'s routine for computing solutions to the OPT_SUBGRAPH problem has to be called as follows:

[solutions,optval]=optsubgraph(SigProject,scenarios,numsol,solver)

Input arguments:

- *SigProject*: a *SigProject* variable as obtained from *loadSigProject* (described in section 4).
- *scenarios*: specifies the set of experiments (scenarios) to be considered for network optimization. In the standard case you will consider all experiments, you can then set *scenarios*=1:SigProject.numexp (this is also the default case if *scenarios* was not defined).
- *numsol*: How many optimal solutions are to be computed (default:1).

- *solver*: Which solver shall be used. *solver*=1 → CPLEX, *solver*=2 → GLPK. If no solver was specified, CPLEX will be assumed as default. (For installation of the solvers see section 3).

The function delivers *numsol* many optimal solutions to the OPT_SUBGRAPH problem which are stored in the columns of the returned array *solutions*. The latter variable has thus dimension q x *numsol* (q = number of network edges). Each of these solutions provides a subgraph of the network which minimizes the mismatch between measured and predicted node states in the perturbation experiments. Each solution contains 1's and 0's. All 1's indicate edges that are REMOVED from the network, hence, the zeros indicate the remaining edges. The i-th row of *solutions* corresponds to the i-th edge in the network (the name of this edge is stored in *SigProject.reacID(i,:))*. The optimal (minimal) mismatch achieved by the found optimal solutions is stored in *optval*. Note that it may happen that less then *numsol* many optimal MCoS exist, in this case the array *solutions* will obviously contain less columns.

6.4 OPT_GRAPH

An OPT_GRAPH problem usually relates to a given network topology and a *set* of experiments (scenarios). *SigNetTrainer*'s routine for computing solutions to the OPT_GRAPH problem has to be called as follows:

[addededges]=optgraph(SigProject,scenarios,solver)

Input arguments:

- *SigProject*: a *SigProject* variable as obtained from *loadSigProject* (described in section 4).
- *scenarios*: specifies the set of experiments (scenarios) to be considered for network optimization. In the standard case you will consider all experiments, you can then set *scenarios*=1:SigProject.numexp (this is also the default case if *scenarios* was not defined).
- *solver*: Which solver shall be used. *solver*=1 → CPLEX, *solver*=2 → GLPK. If no solver was specified, CPLEX will be assumed as default. (For installation of the solvers see section 3).

The function checks for each addable edge (i.e., an edge which (i) is not contained yet in the graph and (ii) which induces no positive feedback loop) how much the addition of this single edge to the network would improve (reduce) the mismatch with the specified set of scenarios when we apply OPT_SUBGRAPH to this extended network. *optgraph* delivers an array *addededges* specifying in the rows the tested added edges and the resulting optimal values for OPT_SUBGRAPH. *addededges* has four columns: the first column defines the start node of the edge, the second the end node, the third the edge sign and the fourth the value of OPT_SUBGRAPH when the latter routine is applied

to the network with the respective edge added. The array is sorted with respect to the mismatch value in the fourth column (hence, the edges with the best fit come first.) If you would like to know the resulting optimal network(s) of the OPT_SUBGRAPH routine for a particular added edge, then add the edge in the netfile, reload the network project via *loadSigProject* and start the function *optsubgraph* as explained above.
In addition to the returned array *addededges*, a file 'addable_edges.txt' is generated (in the current directory) listing the results for each added edge in the same way as in *addededges*, but here by giving directly the names of the nodes instead of their indices.

Part 2: *SigNetTrainer* for GUROBI

1 Introduction

This second part of the present document serves as a getting started guide for *SigNetTrainer* for GUROBI, a toolbox for interrogating and training signaling networks (represented as interaction graphs) based on experimental data from stimulus-response experiments. The GUROBI version of the toolbox was implemented by I.N. Melas and uses the Integer Linear Programming (ILP) framework presented in the main text of this publication. *SigNetTrainer* is also available as a MATLAB/CPLEX and as a MATLAB/GLPK version (see first part of this document).

Given an interaction graph topology (stored in a file "Network.sif") and a set of perturbation experiments (defined in "inputs.txt") with associated measurements (defined in "measurements.txt"), basically four different problems can be addressed by *SigNetTrainer*:

- (1) SCEN_FIT: Determine a causal explanation for the measured activation changes of readout nodes for one given perturbation scenario. If the measurements are inconsistent with the network topology, find the closest feasible scenario.
- (2) Minimal Correction Sets (MCoS): In case of an inconsistent scenario, what is a minimal set of nodes that need to be corrected to make a single inconsistent scenario consistent.
- (3) OPT_SUBGRAPH: Determine an optimal subgraph of the given network topology that can fit the measurements for a set of scenarios at best.
- (4) OPT_GRAPH: Identify edge candidate(s) whose insertion (in combination with removal of existing edges) would improve the consistency of the graph with respect to a set of experimental scenarios at most.

The first two optimization problems seek to match the network topology with measurements from a single stimulus-response experiment. In contrast, (3) and (4) operate on a set of scenarios and seek to optimize (train) the network structure over all scenarios, either by removing or by adding edges. For the first three problems *SigNetTrainer* also provides enumeration algorithms to find multiple or all solutions that solve the optimization problem equally well (e.g., for problem (3), all optimal subgraphs that minimize the number of inconsistencies between measurements and predictions).

The *SigNetTrainer* package also includes some MATLAB files for network compression and preprocessing purposes (see below). In addition, example files for the EGF study discussed in the Results section of the main text are also provided.

Note that the manual will describe usage of the available algorithms rather than redescribing the theory behind the methods.

If you encounter any technical problems please contact I.N. Melas:
giannis.melas@gmail.com.

2 Files

SigNetTrainer comes with the following files:

C source code:

- **"SigNetTrainer.c"**: C source code; it includes the **main()** function of the toolbox and implements the ILP formulations as presented in the Methods section of the main text.
- **"optimize.c"**: C source code; it implements the ILP formulation and employs it to solve problems (1)–(3). It is the function that calls GUROBI optimizer.
- **"import_data.h"**: h file; it includes functions for importing the signaling data (inputs.txt and measurements.txt files).
- **"data_preprocessor.h"**: h file; it includes functions for generating the inputs.txt and measurements.txt files from a tab delimited file, similar to that exported by the Luminex xMAP 200 system.
- **"import_pathway.h"**: h file; it parses the Network.sif file containing the interaction graph.
- **"observable_controllable.h"**: h file; includes files for preprocessing the interaction graph by removing non-observable and non-controllable parts of it. Non-observable are defined all nodes downstream of which there are no measured signals, thus their activation state cannot be inferred. Non-controllable are defined all nodes upstream of which there are no stimuli, thus their activation state cannot be controlled.
- **"warshall.h"**: h file; implements the Floyd-Warshall algorithm for transitive closure. It is used by "observable_controllable.h" to identify the non-observable and non-controllable parts of the pathway.
- **"Makefile"**: The Makefile for compiling the C code (as included in the GUROBI installation). The user should edit it to include the proper path of the GUROBI installation and C libraries. We provide two (exemplary) versions as used on our system: MakefileMac and MakefileLinux (to use them you would need to rename them to 'Makefile').

A file specifying type and parameters of the optimization problem:

- **"ilp_options.txt"**: see section 4.

MATLAB files: These files are required for (optional) network compression and for determining addable edges needed for the OPT_GRAPH problem (see Installation section for how to use):

- **CNASFNetwork2sif.m**
- **CNAsif2SFNetwork.m**
- **addableEdges.m**
- **compressIG.m**

EGFexample: A directory containing the files for the EGFR/ErbB example studied in the Results section of the main text. You may recompute the results presented therein by using these files as input for *SigNetTrainer* (usage is explained in section 4).

MANUAL: A directory containing this manual.

3 Installation

3.1 System Requirements

Requirements: You definitely need to install GUROBI on your computer and to set the path to this library (we used version 4.6.1). We precompiled executable versions for Mac OS X (SigNetTrainerMac) and Linux (SigNetTrainerLinux) and distribute it with the package (but note that GUROBI needs nevertheless to be installed on your system when using these versions).
If you have a different platform or if these versions do not run on your system, you need to recompile it as explained in the next section.

3.2 Compiling the C code

For recompiling the code you proceed as follows:

- You need a working C compiler. We compiled the ILP code using gcc version 4.2 (under Mac OS X).
- You need a working GUROBI installation; we used version 4.6.1 (under Mac OS X).

- In file **optimize.c**, line 5, add absolute path to "gurobi_c.h" file (file installed to your system by GUROBI installer). For Mac OS X default path is "/Library/gurobi461/mac64/include/gurobi_c.h".
- In file optimize.c, lines 34–38 add absolute path to "observable_controllable.h", "import_data.h", "data_preprocessor.h", "warshall.h" and "import_pathway.h" files.
- In a terminal, navigate to the folder where the ILP code is located and run "`make SigNetTrainer`". Before doing that, please update the "Makefile" regarding the paths / variables for GUROBI and the C compiler (if required).

3.3 MATLAB files

Network compression (optionally) and the generation of a list of addable edges (required for the OPT_GRAPH problem) needs to be done via the two MATLAB script files "compressIG.m" and "addableEdges.m", respectively. These files, together with two further m-files for reading/writing ".sif" files (see below), are also distributed with *SigNetTrainer*. For using them, you first, have to install *CellNetAnalyzer* which can be downloaded (free for academic use) from
http://www.mpi-magdeburg.mpg.de/projects/cna/cna.html
After installation of *CellNetAnalyzer*, go to its main directory, start MATLAB and enter "`startcna(1)`". Change then into the directory of *SigNetTrainer.*

For network compression do the following:

- (1) Load the network from a ".sif" file (regarding sif format see section **??**) by running: "`cnap=CNAsif2SFNetwork('FullNetwork.sif')`". Here, "FullNetwork.sif" is the name of the file describing the initial (uncompressed) graph.
- (2) Use MATLAB cell variables to specifiy the names of the (measured) readout nodes and of the nodes that are perturbed, for example "`readouts={'node1','node4'}`" and "`perturb={'node2','node4'}`".
- (3) Call the actual network compression routine by "`cnapnew=compressIG(cnap,readouts,perturb)`".
- (4) Write the new network file which can then be used by *SigNetTrainer*: "`CNASFNetwork2sif(cnapnew,'Networknew.sif')`"
  Here, "Networknew.sif" is the name of the compressed file later to be used by *SigNetTrainer.*

For computing the list of addable edges you need to run "`addableEdges('Network.sif')`". "Network.sif" is the name of the file describing the interaction graph. After running this command, a new file will be written ("addable_reactions.txt") containing all edges that can be considered for OPT_GRAPH (these edges do not induce a positive feedback loop).

4 Running the ILP code

The *SigNetTrainer* code may be used either for preprocessing the signaling dataset, or for executing the optimization procedure, depending on the user's input.

4.1 The file ilp_options.txt

To use the different functions of *SigNetTrainer* the user has to provide/edit four different files:

The file **"ilp_options.txt"** specifies the parameters and options for the different optimization functions of *SigNetTrainer*:

- Tab-delimited file
- **data_preprocessig**: If set to 1, then the ILP code receives as input a data file (e.g. "data.txt") containing the experimental dataset and prints two files named "measurements.txt" and "inputs.txt", specially formatted to be parsed by the optimizer. Set to 1 only if you want to preprocess raw experimenetal data (see later section for howto), else set to 0.
- **significant_increase** (required only for data preprocessing): Corresponds to the threshold above which the fold increase of the signal, after versus before stimulation, is considered to be significant.
- **significant_decrease** (required only for data preprocessing): Corresponds to the threshold below which the fold decrease of the signal, after versus before stimulation, is considered to be significant.
- **noise_threshold** (required only for data preprocessing): An absolute value below which the signal is considered to be insignificant.
- **mipgap**: The relative optimality GAP for the ILP solver (default value is 1E-06, smallest value is 1E-09).
- **timelimit**: Maximum time after which the ILP solver is stopped.
- **number_solutions**: The maximum number of solutions to be identified by the ILP algorithm. The enumeration version of the respective optimization algorithm is used whenever number_solutions ≥ 2.
- **minimum_corrections**: If set to 1, then the Minimum Corrections Sets (MCoS) are identified.
- **best_scenario_fit**: If set to 1, then optimal sign-consistent solutions are computed (SCEN_FIT procedure). If number_solutions $= 1$, a single solution is computed for all experimental scenarios. If number_solutions ≥ 2, only one scenario may be

defined in “inputs.txt” and “measurements.txt”, and all SCEN_FIT solutions will then be enumerated for this scenario.

- **add_new_reactions**: If set to 1, then *SigNetTrainer* parses a list of addable edges provided in file “addable_edges.txt” and scores them based on how much they improve the goodness of fit to the data if added to the interaction graph (OPT_GRAPH problem).
- **subgraphs**: If set to 1, then the total fitting error over all scenarios is minimized by removing edges from the network structure (OPT_SUBGRAPH problem).

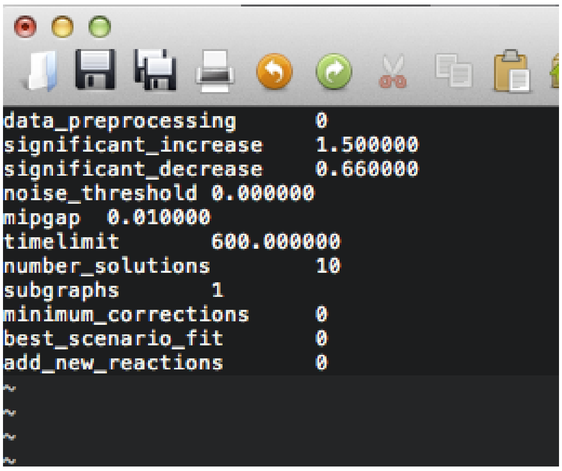

Figure 1: Sample “ilp_options.txt” file

Table 1 summarizes how to set the parameters for the respective optimization problems (single solution and enumeration (enum.) variants).

	SCEN_FIT		MCoS		OPT_SUBGRAPH		OPT_GRAPH
	single sol.	enum.	single sol.	enum.	single sol.	enum.	single sol.
number_solutions	1	≥ 2	1	≥ 2	1	≥ 2	1
subgraphs	0	0	0	0	1	1	0
minimum_corrections	0	0	1	1	0	0	0
best_scenario_fit	1	1	0	0	0	0	0
data_preprocessing	0	0	0	0	0	0	0
add_new_reactions	0	0	0	0	0	0	1
number of scenarios	≥ 1	1	1	1	≥ 1	≥ 1	≥ 1

Table 1: User-defined options and parameters to be set in ilp_options.txt for the different optimization problems (single and enumeration (enum.) variants). The last row shows the number of scenarios that may be defined (in the measurements.txt and inputs.txt files) for the respective problems.

4.2 Other files to be provided/edited by the user

"Network.sif" (defines the interaction graph)

- Tab-delimited file
- Lists the edges (reactions) of the interaction graph row-wise.
- First column contains the start nodes of the respective edge.
- Second column described the edge sign: either it is **positive** ("activation") or **negative** ("inhibition"). Setting a "1" defines a positive, setting a "−1" a negative edge. If set to "2" (or "−2") , then an activation (inhibition) is assumed, and the edge cannot be removed by the algorithm (useful when the user is certain about the edge).
- Third column contains the end node of the respective edge.
- No special characters (except "underscore") are allowed in the names of the reactants or products.
- The names of signaling molecules must correspond to names used as stimuli, inhibitors, or signals in the "data.txt", "inputs.txt", and "measurements.txt" files.

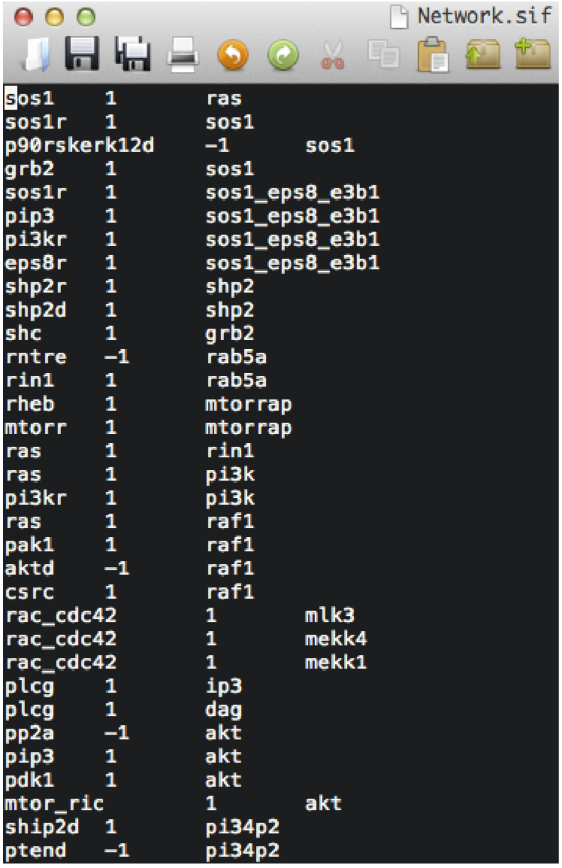
Network.sif

```
sos1	1	ras
sos1r	1	sos1
p90rskerk12d	-1	sos1
grb2	1	sos1
sos1r	1	sos1_eps8_e3b1
pip3	1	sos1_eps8_e3b1
pi3kr	1	sos1_eps8_e3b1
eps8r	1	sos1_eps8_e3b1
shp2r	1	shp2
shp2d	1	shp2
shc	1	grb2
rntre	-1	rab5a
rin1	1	rab5a
rheb	1	mtorrap
mtorr	1	mtorrap
ras	1	rin1
ras	1	pi3k
pi3kr	1	pi3k
ras	1	raf1
pak1	1	raf1
aktd	-1	raf1
csrc	1	raf1
rac_cdc42	1	mlk3
rac_cdc42	1	mekk4
rac_cdc42	1	mekk1
plcg	1	ip3
plcg	1	dag
pp2a	-1	akt
pip3	1	akt
pdk1	1	akt
mtor_ric	1	akt
ship2d	1	pi34p2
ptend	-1	pi34p2
```

Figure 2: Sample “Network.sif” file

“inputs.txt” (defines the perturbations of the experimental scenarios)

- Tab-delimited file
- First row contains the names of the corresponding input (=perturbed) nodes in the columns.
- Subsequent rows correspond to different experimental scenarios, where the columns give the (imposed) values of the perturbed nodes: “1” corresponds to up-regulation of the respective node, “-1” corresponds to down-regulation of the respective node, “0” corresponds to set the respective node to unchanged, and “nan” implies the respective node is not fixed (and thus free) in that scenario.
- No special characters are allowed in the names of the input nodes.
- The names of the input nodes must be consistent wit the corresponding signaling molecules in the “Network.sif” file.

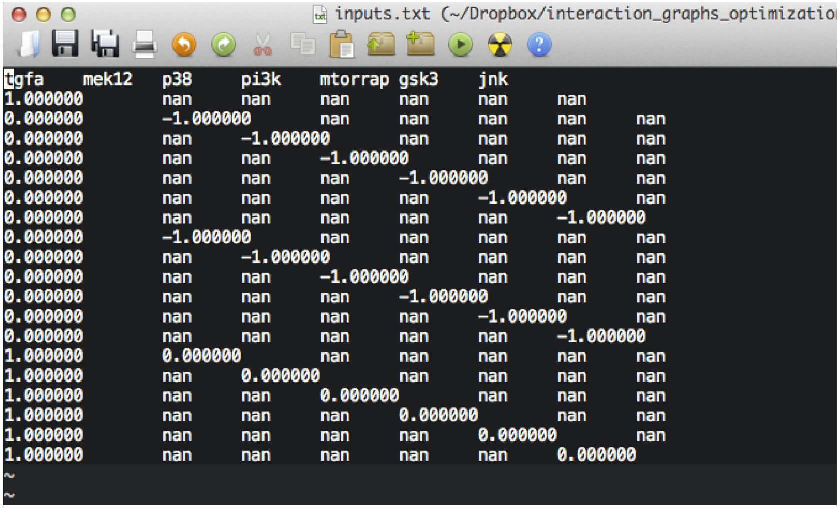


```
tgfa	mek12	p38	pi3k	mtorrap	gsk3	jnk
1.000000	nan	nan	nan	nan	nan	nan
0.000000	-1.000000	nan	nan	nan	nan	nan
0.000000	nan	-1.000000	nan	nan	nan	nan
0.000000	nan	nan	-1.000000	nan	nan	nan
0.000000	nan	nan	nan	-1.000000	nan	nan
0.000000	nan	nan	nan	nan	-1.000000	nan
0.000000	nan	nan	nan	nan	nan	-1.000000
0.000000	-1.000000	nan	nan	nan	nan	nan
0.000000	nan	-1.000000	nan	nan	nan	nan
0.000000	nan	nan	-1.000000	nan	nan	nan
0.000000	nan	nan	nan	-1.000000	nan	nan
0.000000	nan	nan	nan	nan	-1.000000	nan
0.000000	nan	nan	nan	nan	nan	-1.000000
1.000000	0.000000	nan	nan	nan	nan	nan
1.000000	nan	0.000000	nan	nan	nan	nan
1.000000	nan	nan	0.000000	nan	nan	nan
1.000000	nan	nan	nan	0.000000	nan	nan
1.000000	nan	nan	nan	nan	0.000000	nan
1.000000	nan	nan	nan	nan	nan	0.000000
~
~
```

Figure 3: Sample "inputs.txt" file

"measurements.txt" (specifies the resulting node changes (up, down, neutral) in the experiments)

- Tab-delimited file
- First row contains the names of the measured signals (in the columns).
- Subsequent rows correspond to different experimental scenarios, columns specify the measured signals: "1" corresponds to up-regulation, "-1" corresponds to down-regulation, "0" corresponds to unchanged signal, and "nan" implies the respective signal is not measured in that scenario.
- No special characters are allowed in the names of the measured signals.
- The names of the measured signals must be consistent with the corresponding signaling molecules in the "Network.sif" file.

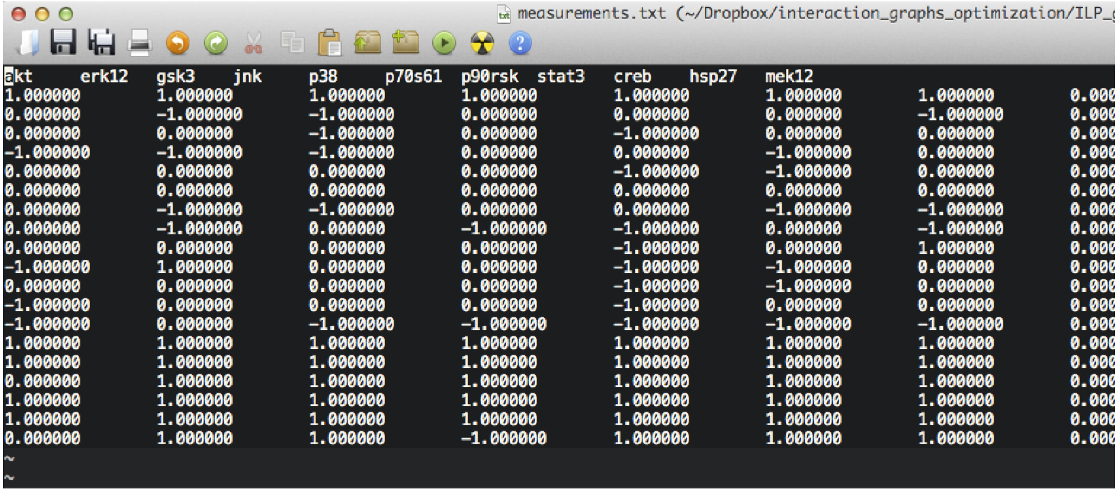

measurements.txt (~/Dropbox/interaction_graphs_optimization/ILP_

```
akt	erk12	gsk3	jnk	p38	p70s61	p90rsk	stat3	creb	hsp27	mek12
1.000000	1.000000	1.000000	1.000000	1.000000	1.000000	1.000000	0.000
0.000000	-1.000000	-1.000000	0.000000	0.000000	0.000000	-1.000000	0.000
0.000000	0.000000	-1.000000	0.000000	-1.000000	0.000000	0.000000	0.000
-1.000000	-1.000000	-1.000000	0.000000	0.000000	-1.000000	0.000000	0.000
0.000000	0.000000	0.000000	0.000000	-1.000000	-1.000000	0.000000	0.000
0.000000	0.000000	0.000000	0.000000	0.000000	0.000000	0.000000	0.000
0.000000	-1.000000	-1.000000	0.000000	0.000000	-1.000000	-1.000000	0.000
0.000000	-1.000000	0.000000	-1.000000	-1.000000	0.000000	-1.000000	0.000
0.000000	0.000000	0.000000	0.000000	-1.000000	0.000000	1.000000	0.000
-1.000000	1.000000	0.000000	0.000000	-1.000000	-1.000000	0.000000	0.000
0.000000	0.000000	0.000000	0.000000	-1.000000	-1.000000	0.000000	0.000
-1.000000	0.000000	0.000000	0.000000	-1.000000	0.000000	0.000000	0.000
-1.000000	0.000000	-1.000000	-1.000000	-1.000000	-1.000000	-1.000000	0.000
1.000000	1.000000	1.000000	1.000000	1.000000	1.000000	1.000000	0.000
1.000000	1.000000	1.000000	1.000000	1.000000	1.000000	1.000000	0.000
0.000000	1.000000	1.000000	1.000000	1.000000	1.000000	1.000000	0.000
1.000000	1.000000	1.000000	1.000000	1.000000	1.000000	1.000000	0.000
1.000000	1.000000	1.000000	1.000000	1.000000	1.000000	1.000000	0.000
0.000000	1.000000	1.000000	-1.000000	1.000000	1.000000	1.000000	0.000
~
~
```

Figure 4: Sample "measurements.txt" file

4.3 Data preprocessing

We implemented an optional data preprocessing which delivers the files "inputs.txt" and "measurements.txt"(described above) in an automatic way from raw data sets. This preprocessing is tailored to the type of data we are normally using (Luminex). Of course, the user may put together these two files on its own if this is more convenient (see previous section 4.2 how to format "measurements.txt" and "inputs.txt").

In order to use the data preprocessing feature of *SigNetTrainer*, you must provide a file "data.txt" with the following structure:

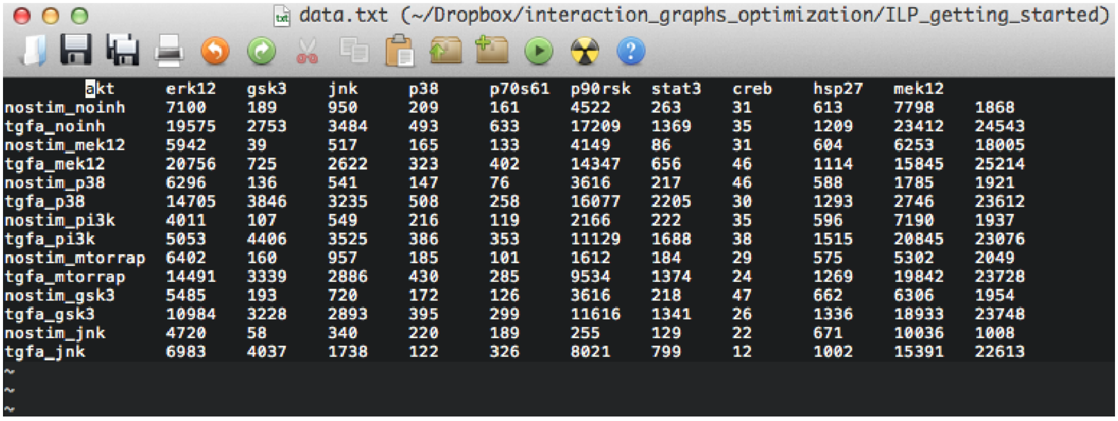

data.txt (~/Dropbox/interaction_graphs_optimization/ILP_getting_started)

```
	akt	erk12	gsk3	jnk	p38	p70s61	p90rsk	stat3	creb	hsp27	mek12
nostim_noinh	7100	189	950	209	161	4522	263	31	613	7798	1868
tgfa_noinh	19575	2753	3484	493	633	17209	1369	35	1209	23412	24543
nostim_mek12	5942	39	517	165	133	4149	86	31	604	6253	18005
tgfa_mek12	20756	725	2622	323	402	14347	656	46	1114	15845	25214
nostim_p38	6296	136	541	147	76	3616	217	46	588	1785	1921
tgfa_p38	14705	3846	3235	508	258	16077	2205	30	1293	2746	23612
nostim_pi3k	4011	107	549	216	119	2166	222	35	596	7190	1937
tgfa_pi3k	5053	4406	3525	386	353	11129	1688	38	1515	20845	23076
nostim_mtorrap	6402	160	957	185	101	1612	184	29	575	5302	2049
tgfa_mtorrap	14491	3339	2886	430	285	9534	1374	24	1269	19842	23728
nostim_gsk3	5485	193	720	172	126	3616	218	47	662	6306	1954
tgfa_gsk3	10984	3228	2893	395	299	11616	1341	26	1336	18933	23748
nostim_jnk	4720	58	340	220	189	255	129	22	671	10036	1008
tgfa_jnk	6983	4037	1738	122	326	8021	799	12	1002	15391	22613
~
~
~
```

Figure 5: Sample "data.txt" file

"data.txt"

- Tab-delimited file
- Rows correspond to different experimental scenarios, columns correspond to measured signals.
- First column contains a description for the corresponding scenarios in the following format: "stimuli_inhibitor". Where, "stimuli" is the stimuli introduced in the current scenario, "inhibitor" is the inhibitor (or knock out) introduced in the current scenario.
- First row contains the names of the corresponding signals.
- No special characters are allowed in the labels of the scenarios or the signals.
- Names of the signals, stimuli or inhibitors must be the same with the ones included in the interaction graph.
- The remaining entries are floating point numbers that correspond to activation values.

After having compiled the C code, one may then run: "`./SigNetTrainer data.txt`". "SigNetTrainer.c" will read the "ilp_options.txt" file, containing user-defined options for data preprocessing (see above), and the data file itself ("data.txt") containing the experimental dataset as described above. The two files named "measurements.txt" and "inputs.txt" will then be generated.

4.4 Running the optimization procedure

In a terminal, after having compiled the C code run:

"`./SigNetTrainer Network.sif inputs.txt measurements.txt`".

"SigNetTrainer.c" will read the user-defined options from "ilp_options.txt". Afterwards, it reads **(i)** a file containing the interaction graph (e.g. "Network.sif", see example file in figure **??**), **(ii)** a file containing the experimental design (e.g. "inputs.txt", see example in figure **??**), **(iii)** a file containing the measured data (e.g. "measurements.txt", see example in figure **??**), and then returns the optimization results "objective_value.txt", "reactions_out.txt" (or "interventions_out.txt"), "network_out.txt", predicted values for the signaling molecules "predictions_out.txt" and auxiliary files "species_indices.txt", "solutions_history.txt", "reactions_indices.txt".
Importantly, for the OPT_GRAPH procedure you need to give in addition a fourth file ("addable_edges.txt") specifying the edges that can potentially be added, thus you need to call:

"`./SigNetTrainer Network.sif inputs.txt measurements.txt addable_edges.txt`".

Output files

- **"objective_value.txt"**: The fitting error of the interaction graph w.r.t. the measured data.
- **"reactions_out.txt"**: A list containing all edges in the interaction graph and an identifier showing whether the edge was removed during the optimization procedure or not. Tab delimited file. The first column contains the edge IDs. The third column, and every column after that, contains two numbers separated by a comma character. The first number in each column is used if the corresponding edge is an activation, the second number is used if the corresponding edge is an inhibition. An entry of 1.0 denotes the corresponding edge was removed by the ILP, an entry of 0.0 denotes the edge was conserved in the solution. Every column corresponds to a different solution in the solution pool (if "number_solutions" ≥ 2 in the "ilp_options.txt" file).
- **"network_out.txt"**: The network structure in .dot format ready to be parsed by graphviz.
- **"predictions_out.txt"**: A table containing the activation states of measured signals as predicted by the ILP algorithm. Rows correspond to different experimental scenarios, columns correspond to measured signals. First row contains the names of the measured signals. The rest of the entries correspond to the predicted activation values of the signals. "1" corresponds to up-regulation of the respective signal, "-1" corresponds to down-regulation of the respective signal, "0" corresponds to predicting the respective signal as inactive.
- **"species_indices.txt"**: A list containing all signaling nodes (i.e. species) of the interaction graph and their corresponding ID.
- **"reactions_indices.txt"**: A list containing all edges of the interaction graph and their corresponding ID.
- **"solutions_history.txt"**: Includes the same information as "network_out.txt" formatted differently. Includes a list of the $y_{i,s}$ variables (see Methods section of the main text), where every column corresponds to a different solution in the solution pool. Every column numbers $2N$ rows, where N = Number_of_reactions. The first N rows correspond to the $y^+_{i,s}$ variables, the last N rows correspond to the $y^-_{i,s}$ variables.
- **"interventions_out.txt"**: Is generated if "minimum_corrections" = 1 in the "ilp_options.txt" file. A list containing all species (i.e. signaling nodes) and experimental scenarios in the interaction graph and an identifier showing whether each species was perturbed (and how) for that given experiment. Tab delimited file. The first column contains the scenario ID. The second column contains the species ID. The third column, and every column after that, contains two numbers separated by a comma character. The first number in each column corresponds to

positive perturbation (up-regulation) of the respective node, the second number corresponds to negative perturbation (down-regulation) of the respective node. An entry of 1.0 denotes the corresponding node was perturbed by the ILP, an entry of 0.0 denotes the node was not perturbed. Every column corresponds to a different solution in the solution pool (if "number_solutions" ≥ 2 in the "ilp_options.txt" file).

- **"edges_scored.txt"**: Is generated if "add_new_reactions" = 1 in the "ilp_options" file. A list containing all edges specified in "addable_edges.txt" and the fitting error that results from OPT_SUBGRAPH if this (single) edge was added.

As not all output files are relevant for each of the four different problems, Table 2 gives an overview which files should be considered for the respective problem.

	SCEN_FIT	MCoS	OPT_SUBGRAPH	OPT_GRAPH
objective_value	×	×	×	
reactions_out			×	
network_out			×	
predictions_out	×		×	
species_indices		×	×	
reactions_indices			×	
solutions_history			×	
interventions_out		×		
edges_scored				×

Table 2: Output files to be considered for the four different problems.
