# Supplementary material for: Detecting and Removing Inconsistencies between Experimental Data and Signaling Network Topologies Using Integer Linear Programming on Interaction Graphs
Source: PLoS Comput Biol. 2013 Sep 5;9(9):e1003204. doi: 10.1371/journal.pcbi.1003204 (PMC3764019; doi:10.1371/journal.pcbi.1003204)
Supplement: Text S2 — Sensitivity analysis of the SCEN_FIT solutions with respect to the chosen discretization thresholds. (PDF) [file pcbi.1003204.s006.pdf]

Sensitivity analysis of the SCEN_FIT solutions with respect to the chosen discretization thresholds

The ILP formulations presented herein receive as input the interaction graph and signaling data and solve the SCEN_FIT, MCoS, OPT_SUBGRAPH and OPT_GRAPH problems. As described in the main text, the data have to be imported in a discretized form, assuming the values “-1” (for down-regulation), “0” (for unchanged), or “1” (for up-regulation). To this effect, the fold change of the signal in the stimulated versus the unstimulated state is used. If the fold change is above a certain threshold named “significant increase”, the respective signal is considered to be up-regulated; if the fold change is below “significant increase” but above “significant decrease”, then the signal is considered to be inactive, and if the fold change is below “significant decrease”, then the signal is considered to be down-regulated. The values for “significant increase” and “significant decrease” used in the paper are 1.5 and 0.667, respectively. Herein, to test the impact of the chose thresholds on the ILP formulation, we perform sensitivity analysis of the SCEN_FIT results with respect to these thresholds.

We screen 80 different values for “significant increase” from 1.025 to 3.025, and 40 values for “significant decrease” from 0.1 to 1.0 and plot the resulting cumulative fitness error of the SCEN_FIT problems (over all 16 scenarios) in Figure S2. The larger “significant decrease” and the smaller “significant increase” becomes, the more over-responsive the discretized dataset is (i.e., even the smallest change in the activation of a signal is considered significant). We observe that there is a relatively large range of “significant decrease” in $[0.2, 0.7]$ and “significant increase” in $[1.5, 1.9]$ where the total fitness error assumes its lowest value (between 40 and 50). Outside that area, the fitness error increases rapidly. The thresholds used in the EGFR/ErbB study (0.667 and 1.5, respectively) are inside that range and result in a total fitness error of 45 (see Figure 3 in the main text).
