# Supplementary material for: Detecting and Removing Inconsistencies between Experimental Data and Signaling Network Topologies Using Integer Linear Programming on Interaction Graphs
Source: PLoS Comput Biol. 2013 Sep 5;9(9):e1003204. doi: 10.1371/journal.pcbi.1003204 (PMC3764019; doi:10.1371/journal.pcbi.1003204)
Supplement: Text S3 — Systematic evaluation of ILP runtimes with respect to problem size. (PDF) [file pcbi.1003204.s007.pdf]

Systematic evaluation of ILP runtimes with respect to problem size

The ILP formulations presented in this paper address the detection and removal of inconsistencies between experimental data and interaction graphs by four fundamental optimization problems: SCEN_FIT, MCoS, OPT_SUBGRAPH and OPT_GRAPH. The runtimes of these optimization problems in the EGFR/ErbB case study were not greater than a few seconds. However, since ILPs are NP-hard, we tested the runtime behavior of the proposed formulations more systematically by means of benchmarks to provide information on scalability and the ability of the algorithms to tackle larger, more complex problems.

Here we discuss how the formulations for all four problems (SCEN_FIT, MCoS, OPT_SUBGRAPH and OPT_GRAPH) perform in terms of runtime against networks and data of variable size (see Figure S3). Four networks (all being variants of the EGFR/ErbB network) were used for the benchmarks numbering 18, 32, 42 and 67 edges respectively. The smallest one (18 edges) is the compressed network presented and analyzed in the main text (Figure 4B). The largest one (67 edges) is the EGFR network without any compression, but after removing non-observable and non-controllable parts of it (Figure 4A). The other two (32 and 42 edges) are variants of the EGFR network with partial compression. All four networks include the same input and measured nodes. Moreover, four different datasets were used: (i) the original EGFR dataset used throughout this paper, numbering 11 signals under 16 experimental scenarios, named "EGFR data"; (ii) a random dataset of equal size (11 signals under 16 experimental scenarios), named "random data"; (iii) a random dataset with twice the number of scenarios but same number of signals (11 signals under 32 scenarios), named "more scenarios"; (iv) a random dataset with the same number of scenarios, but with measurements for all nodes apart from the input nodes, named "more signals" (the network with the 18 edges has 13 signals, the one with the 32 edges has 18 signals, the one with the 42 edges has 23 signals, the one with the 67 edges has 38 signals). All four problems were solved (search for a single solution as well as enumeration of all solutions) under all data and network combinations. For every run, the CPU time, number of variables, number of constraints, and number of solutions are reported. Results are shown in Figure S3.

With respect to SCEN_FIT, the formulation was applied to each scenario of the respective dataset, then CPU time and statistics were averaged across all scenarios of the same problem size. Figure S3 shows the mean values. All benchmarks completed in 0.01 seconds or less. The corresponding ILPs numbered for the EGFR data from 179 variables and 369 constraints (mean values) for the compressed network (18 edges) up to 597 variables and 1254 constraints for the uncompressed network (67 edges), thus showing a significant advantageous effect of the compression techniques. These values were slightly increased in the "more signals" dataset. Note that the statistics for "single solution" and "enumeration" are identical since the algorithm could not identify more than one optimal solution. This shows that the SCEN_FIT problem seems to be well constrained, at least in the considered datasets. The "more scenarios" dataset was not interrogated since SCEN_FIT is applied on a single scenario at a time, thus the number of scenarios does not affect the average CPU time or other statistics.

With respect to MCoS, the formulation was again applied to each scenario of the respective dataset, then CPU time and statistics were averaged across all scenarios (in similar fashion to SCEN_FIT). All benchmarks completed within a second apart from the enumeration of a dozen solutions for the uncompressed network under "random data", which completed in 3.36 seconds in the average. In contrast to SCEN_FIT, the enumeration of solutions returned more than one solution. As expected, the larger the interrogated network, the more MCoS solutions were obtained. The number of variables was close to that of SCEN_FIT for the single solution, ranging from 207 variables and 370 constraints for the compressed network (18 edges) to 657 variables and 1255 constraints for the uncompressed network (67 edges). The number of constraints increases slightly in the enumeration benchmarks, since in every solution after the first one extra constraints are introduced to exclude the previous solutions from the current run. As for SCEN_FIT, the "more scenarios" dataset was not interrogated since MCoS is applied on a single scenario at a time, thus the number of scenarios does not affect the average CPU time or other statistics.

With respect to single OPT_SUBGRAPH solutions, all benchmarks completed within a few seconds with only one exception, the optimization of the uncompressed network to the "more scenarios" data (needing

$\sim$ 1400 seconds). Small runtimes were observed for the original EGFR data used in the paper, longer time was required for the "random" and the "more signals" data, and substantially longer runtime was required for the "more scenarios" data. As expected, runtime also increased with network size. The number of variables and constraints for the "EGFR data" ranges from 2594 variables and 5612 constraints for the compressed network up to 8547 variables and 18988 constraints for the uncompressed network. Regarding the enumeration of alternate OPT_SUBGRAPH solutions, only the compressed network could be successfully optimized to "EGFR data", "random data" and "more scenarios". The enumeration under "more signals" aborted after reaching the maximum allowed number of solutions (1000) indicating that a huge number of optimal solutions exists. The enumeration of solutions for all other networks did not complete either because the maximum number of solutions (1000) or the time limit (64,000 seconds) was exceeded. The number of constraints increases significantly as more solutions are identified, since in every solution after the first one an extra constraint is introduced to exclude previous solutions from the current run (for instance, the number of constraints increases from 5612 (first run) to 5828 (last run) when enumerating the OPT_SUBGRAPH solutions of the compressed network under "random data"). Generally, the OPT_SUBGRAPH benchmarks give us valuable insight on the performance of the proposed formulation. First, as expected, it is evident from the large number of variables and constraints compared to SCEN_FIT and MCoS formulations that OPT_SUBGRAPH is a more complex, computationally demanding problem as it optimizes over all scenarios instead of a single one. Second, the performance of the algorithm varies from case to case even for problems of the same size. For example, the optimization of the uncompressed network (single solution) to "random data" and "EGFR data" requires the same number of variables and constraints, yet a significant increase in runtime is observed under "random data" compared to "EGFR data". This may be attributed to the fact that "EGFR data" are closer to the predictions of the interaction network, or differently put, that "random data" have more internal conflicts than "EGFR data". Since "random data" were generated in random manner they fit the interaction network much worse than the experimental "EGFR data". Thus, under "EGFR data" the algorithm initiates the optimization procedure with less fitting error (closer to the optimum) and terminates faster. In similar fashion, finding a single optimal OPT_SUBGRAPH for the uncompressed network exposed to the "more scenarios" data is even more unfavorable, since the extra scenarios create not only additional variables and constraints but also more internal conflicts and a larger fitting error. It is noteworthy that finding a single solution in the uncompressed network required close to 1400 seconds compared to 10.62 seconds under "random data" and 0.22 seconds under "EGFR data". Moreover, comparison of the "more data" and "more scenarios" cases indicates that expanding the experimental dataset with respect to the number of signals does not affect the performance of the formulation as much as expanding it with respect to the number of scenarios does. Finally, our network compression procedure not only decreases the network size, thus facilitating the optimization procedure, but also substantially decreases the number of optimal solutions. The compressed network numbers only 6 optimal solutions under "EGFR data", while even a partially compressed network with 32 edges numbers more than 1000 solutions (the algorithm stopped after having found this maximal number). The same behavior applies for the "random data" and "more scenarios" cases. A low number of optimal solutions indicates an adequately constrained optimization problem that yields utilizable results. Note that a solution of OPT_SUBGRAPH found in the compressed network often corresponds to multiple solutions in the uncompressed versions explaining why the number of solutions in the uncompressed versions can be much larger.

With respect to OPT_GRAPH, the runtimes are significantly increased compared to single solutions of OPT_SUBGRAPH, since for a complete run the OPT_SUBGRAPH problem has to be solved for all addable candidate edges to calculate how the fitting error is affected by the addition of each single edge. The number of addable edges to screen is increased with the networks size: for the compressed network, 298 addable edges exist; for the partially compressed network with 32 edges we have 536 addable edges; for the network with 42 edges 860 addable edges exist, and for the uncompressed network there are 2351 addable edges. Since we are only interested in the effects of the addable edges on the fitting error, only a single solution of the OPT_SUBGRAPH problem needs to be computed for each edge. OPT_GRAPH successfully completed for all networks under the "EGFR data" and for several networks under "random data" and "more signals". For "EGFR data" the runtimes were low (even the uncompressed network was solved in little over an hour) compared to "random data" and "more signals" that required up to 10 hours. For "more scenarios" the optimization could only be finished for the compressed network within the time limit (64,000 seconds). The OPT_GRAPH benchmarks validate the conclusions from before: (i)

the performance of the formulation varies even for problems of the same size (the runs under "random data" required more time to complete compared to "EGFR data"), and (ii) in problems with large and noisy datasets, the runtime increases significantly and testing of all edges was not possible within the given time limit.

Overall, most of the benchmarks completed within a few seconds and only the enumeration of solutions for some larger OPT_SUBGRAPH and OPT_GRAPH problems could not complete either due to memory limitations or due to run time out. However, all problems (including OPT_GRAPH and OPT_SUBGRAPH) were successfully tackled (both single solution and enumeration) if the compression algorithm was first applied to the network in a preprocessing step. Our compression algorithm removes nodes and edges whose presence in the network cannot be distinguished based on the data at hand, decreasing the networks size and facilitating the optimization procedure. Moreover, based on the OPT_SUBGRAPH and OPT_GRAPH benchmarks, the performance of the formulation was found to vary from case to case even for problems of the same size (the runs under "random data" required more time to complete compared to "EGFR data"), implying that noisy data with many conflicts with respect to the interaction network are more difficult to interrogate. All calculations were done on a relatively "simple" PC with a 2.2GHz intel quad core i7 CPU (only a single core was used) and 4GB 1333MHz DDR3 memory. Hence, using, for example, a computer cluster could increase the feasible problem size extensively.
